# Comparative analysis of microbial succession and biochemical changes in cigar tobacco filler and wrapper leaves during fermentation

**DOI:** 10.3389/fmicb.2026.1922631

**Published:** 2026-10-02

**Authors:** Zhaoliang Geng, Bin Cai, Yanqi Hu, Beisen Lin, Tongjing Yan, Huajun Gao, Abdullah Khan, Xinghua Ma

**Affiliations:** 1 Hainan Provincial Branch of China National Tobacco Corporation, Haikou, China; 2 China Tobacco Shandong Industrial Co., Ltd., Jinan, China; 3 Key Laboratory of Tobacco Biology and Processing, Ministry of Agriculture and Rural Affairs, Tobacco Research Institute of Chinese Academy of Agricultural Sciences, Qingdao, China

**Keywords:** cigar tobacco, fermentation, filler leaves, microbial succession, volatile compounds, wrapper leaves

## Abstract

Bulk fermentation of cigar tobacco leaves couples microbial succession and chemical transformations; however, it remains unclear whether commercially distinct filler and wrapper leaves follow different ecological and biochemical trajectories under identical industrial conditions. Using 16S rRNA and ITS amplicon sequencing, together with conventional chemical indices and volatile profiling, we tracked filler and wrapper leaves (two commercial cultivars, variety 204 and variety 108) through 0, 10, 18, 49, and 89 days of industrial bulk fermentation. The results revealed that reducing sugars declined by 63% in filler leaves (0.57 to 0.21%) and 66% in wrapper leaves (0.59 to 0.20%), whereas alkaloid dynamics differed between materials: filler leaves showed a transient mid-fermentation rise before decline, while wrapper leaves declined from the outset. Bacterial communities shifted from Proteobacteria-dominated early assemblages toward increased Firmicutes during later fermentation, with *Acinetobacter, Staphylococcus* and *Pseudomonas* as major genera. Fungal communities were dominated primarily by *Ascomycota*, with *Aspergillus* prevalent across fermentation and *Papiliotrema* becoming more prevalent at later stages. Bray-Curtis based ordination showed structured, directional succession in both bacterial and fungal communities, and fermentation time explained substantially more community variation than material type (bacteria: R^2^ = 0.57 vs. 0.04; fungi: R^2^ = 0.42 vs. 0.08). Correlation, redundancy and network analyses linked both bacterial and fungal community variation to sugars, nitrogenous compounds, alkaloids, and volatile classes, although these associations remain correlative rather than causal. By directly comparing filler and wrapper leaves under identical industrial fermentation environment, this study highlights that commercially distinct cigar materials follow divergent ecological and biochemical trajectories, providing a process-resolved basis for fermentation monitoring and material-specific quality management.

## 1 Introduction

Many plant-derived raw materials, including tea and tobacco (Huang et al., 2010), require post-harvest bioprocessing, including fermentation or aging, to improve sensory quality, reduce undesirable constituents, and improve usability (Lawyer et al., 2019; Long et al., 2020). During fermentation, macromolecular and undesirable constituents are degraded or transformed into smaller and more desirable compounds (Lang et al., 2025; Zhou et al., 2024), thereby improving flavor quality, processability, and consumer acceptability (Marco et al., 2017). However, fermentation remains a complex biochemical and ecological process involving tightly coupled abiotic reactions, endogenous enzymes, and microbial activity that are still incompletely resolved (Adebo and Medina-Meza, 2020). Clarifying these transformations is important for improving the quality and stability of fermented plant products (Li et al., 2024).

In tobacco processing, fermentation is an indispensable step for improving leaf quality, aroma, and combustibility (Frankenburg, 1950). Unfermented tobacco leaves typically exhibit harshness, unpleasant odor, and insufficient aroma, rendering them unsuitable for cigar production (Yang et al., 2018). During fermentation, a series of biochemical transformations mediated by endogenous enzymes and leaf-associated microorganisms promote the degradation or transformation of alkaloids, nitrogenous compounds, and polyphenols (Liu et al., 2011), while facilitating the formation of volatile aroma compounds such as esters, alcohols, aldehydes, and acids (Wan et al., 2017). These biochemical conversions darken the leaf color, reduce irritation, and shape the flavor profile desired in high-quality tobacco (Hu et al., 2022). Among the commonly used methods, stack (bulk) fermentation relies on the self-heating of moistened leaves piled into heaps, where temperature may reach 55–65 °C due to intensified biological and physicochemical activity (Li et al., 2020). Periodic turning is used to promote uniform fermentation and to prevent overheating. Under industrial bulk fermentation conditions, temperatures are commonly maintained within 35–50 °C for several weeks (Wang et al., 2014), optimal conditions vary with cultivar., maturity, and production region, indicating that fermentation parameters must be fine-tuned to specific raw materials (Wu et al., 2021).

The assembly and succession of microbial communities play a pivotal role in tobacco fermentation (Zhang L. Y. et al., 2024). Microorganisms colonize leaf surfaces early in the process and then shift dynamically in response to nutrient composition, temperature, moisture, and other processing-related filters, together with deterministic and stochastic processes such as selection, dispersal, diversification, and ecological drift, consistent with general ecological and evolutionary frameworks developed for fermented-food microbiomes (Wang F. et al., 2022; Louw et al., 2023). The chemical and ecological characteristics of the raw material strongly influence microbial selection because variation in substrate type, concentration, and accessibility shapes the metabolic landscape available for microbial growth (Sha et al., 2025). Even with the same plant crop, differences among leaf categories or cultivation environment can alter chemical composition and thereby modify microbial colonization patterns (Zhang et al., 2023). Fermentation is thus a time-dependent ecological process in which successive microbial populations are associated with stepwise changes in carbohydrates, proteins, alkaloids, and volatile compounds (Wang M. et al., 2022). Environmental parameters, including aeration and humidity, further modulate microbial composition and activity (Zheng et al., 2022). Cigar tobacco fermentation typically occurs under moderate pile conditions that support a diverse range of microorganisms, making it a useful system for studying how community succession accompanies biochemical quality formation (Yin et al., 2025). This ecological framework is not unique to Chinese domestic tobacco: comparative surveys of cigars from major producing regions, including the Caribbean, South America, and Southeast Asia, show that bacterial community composition, rather than fungal composition, differentiates cigars by geographic origin, with Staphylococcus dominant in Americas-origin cigars and *Bacillus* dominant in Southeast Asian cigars (Liu et al., 2022), indicating that the fermentation-driven microbial dynamics examined here connect to a broader, internationally documented pattern of cigar-leaf microbial ecology.

Previous studies on tobacco fermentation have mainly described microbial community composition, general chemical changes, or fermentation trajectories of individual materials. Less attention has been given to whether commercially distinct cigar materials, such as filler and wrapper leaves, follow different ecological and biochemical trajectories when fermented under the same processing environment. This distinction is important because filler and wrapper leaves differ in structure and initial chemical composition, which may influence substrate accessibility, microbial selection, and the timing of aroma development. Existing comparisons among wrapper, binder, and filler cigar tobaccos have largely characterized microbial and flavor differences at a single, typically post-fermentation, timepoint (Zhang M. et al., 2024), and our own previous work similarly compared wrapper and filler leaves of different varieties and origins mainly after fermentation was complete (Geng et al., 2025); how these two materials diverge across the fermentation timeline itself, rather than only at its endpoint, remains largely unresolved. In this study, we compared filler and wrapper cigar tobacco leaves fermented at the same location under the same bulk fermentation regime and integrated amplicon sequencing, conventional chemical indices, and volatile compound profiling across five stages. The objectives were to (i) characterize temporal successions of bacterial and fungal communities, (ii) compare the ecological trajectories of filler and wrapper leaves under shared fermentation conditions, and (iii) identify statistically supported associations between microbial community variation and chemical transformation. By focusing on comparative fermentation trajectories rather than a single material alone, this study provides a process-level framework for monitoring cigar fermentation and may also inform controlled fermentation of other plant-derived agricultural materials in which substrate heterogeneity shapes microbial succession and quality formation.

## 2 Materials and methods

### 2.1 Experimental location and details

The cigar tobacco leaves used in this experiment were grown in Danzhou, Hainan Province, China, in 2022. Cigar tobacco variety 204 was used as the filler (F) leaf sampling, whereas variety 108 was used as the wrapper (W) leaf samples. Because these two materials represent commercially relevant filler and wrapper categories used in practice, the comparison was designed to evaluate fermentation differences between commercial filler and wrapper materials under identical processing conditions rather than to isolate genotype-independent leaf functional effects. After air drying, the leaves were transported to the Tunchang Experimental Station of Haikou Cigar Research Institute for conventional bulk fermentation. About 50 kg of leaves from each material were randomly selected and fermented in separate piles. Each pile measured 1.5 m in width, 1.2 m in height, and 2.0 m in length. Daily temperature and humidity of wrapper and filler leaves over the 90-day fermentation are presented in Supplementary Figure S1. Samples were collected before fermentation (day 0) 10, 18, 49, and 89 days during fermentation. To standardize temporal sampling, samples were collected each time from the center of the pile at approximately two-third of the pile height, representing the core fermentation zone. This strategy improves consistency across time points, although it did not fully capture within-pile heterogeneity. Samples were immediately sealed after air removal and stored at −20 °C until analysis. The piles were turned after each sampling event. Microbial community composition, conventional chemical indices, and volatile compounds were analyzed using three independent biological replicates for each material and time point, taken as independent sub-samples from the single fermentation pile established for each material. Because each material was fermented as one 50 kg pile rather than in multiple independent piles, these replicates capture within-pile sampling variation rather than pile-to-pile variation.

### 2.2 Biochemical profiling

#### 2.2.1 Basic chemical compounds detection

Key chemical constituents, including reducing sugars, total sugars, total nitrogen, potassium (K) and chlorine (Cl), were measured using a continuous-flow analyzer (Futura-II, Alliance, FR) as previously described by Wang F. et al. (2022). In addition, a separate gas chromatography–mass spectrometry (GC–MS) based chemical profiling was performed. Samples were extracted with acetonitrile using a QuEChERS kit and analyzed on a DB-5MS column. The GC oven temperature was programmed from 60 °C to 290 °C using defined holding times and ramp rates. Compounds were identified by comparison with the NIST and Wiley mass spectral libraries and semi-quantified relative to the internal standard phenylethyl acetate.

#### 2.2.2 Volatile aroma compounds detection

Volatile compounds were analyzed using headspace solid-phase microextraction coupled with gas chromatography–mass spectrometry (HS-SPME-GC–MS; Trace GC Ultra-DSQ II, Thermo Fisher Scientific, Austin, TX, USA), following a previously described method with minor modifications (Zhang et al., 2023). Briefly, 0.5 mL of sample was mixed with 2.0 mL of deionized water (ddH_2_O), 1.5 g of sodium chloride (NaCl), and 10 μL of an internal standards solution containing methyl octanoate and 2-octanol in a 15 mL headspace vial. The vial was sealed with a screw cap fitted with a silicone septum and pre-equilibrated in a thermostatic water bath at 60 °C with magnetic stirring for 15 min. A DVB/CAR/PDMS fiber (Supelco, Bellefonte, PA, USA) was then exposed to the headspace for 40 min to adsorb volatile compounds. After extraction, the fiber was immediately desorbed in the GC injector for 5 min. Chromatographic separation was achieved using an HP-Innowax capillary column (30 m × 0.25 mm × 0.25 μm; Agilent J&W, Santa Clara, USA). Helium was used as the carrier gas at a constant flow rate of 1.0 mL min^−1^ under splitless mode. The injector and transfer line temperatures were both set at 250 °C. Mass spectrometric detection was conducted using electron impact ionization at 70 eV, with a scan range of m/z 35–400 and an ion source temperature of 230 °C. The GC oven program was as follows: initial temperature of 40 °C for 5 min, increased to 100 °C at 4 °C/min, then to 220 °C at 6 °C/min and held for 5 min. Compounds were identified by comparison with the National Institute of Standards and Technology (NIST/EPA/NIH Mass Spectral Library (NIST 05), 2005) library and by confirmation of Kovats retention indices (RI) calculated using a homologous series of C8-C20 n-alkanes. Volatile components were semi-quantified using internal-standard-based peak area normalization (Zhang et al., 2023).

### 2.3 Leaf DNA extraction experiment

The composition of leaf-associated microbial communities was examined using a high-throughput amplicon metabarcoding strategy. Bacterial diversity was assessed by amplifying V5-V7 hypervariable regions of the 16S rRNA gene, which provide reliable taxonomic resolution. Amplification employed primer pair 799F (5′-AACMGGATTAGATACCCKG3′) and 1193R (5′-ACGTCATCCCCACCTTCC-3′) (Liang et al., 2024). Fungal diversity was characterized by targeting the Internal Transcribed Spacer 2 (ITS2) region using primers ITS_KYO2-F (5′-GATGAAGAACGYAGYRAA-3′) and ITS4-R (5′-TCCTCCGCTTATTGATATGC-3′), which provided broad fungal coverage (Chen et al., 2020). Amplicon libraries were sequenced on the Illumina HiSeq platform to generate 2 × 250 bp paired-end reads, ensuring sufficient overlap for high-quality assembly. Detailed DNA extraction and amplification procedures are described below.

#### 2.3.1 DNA extraction, PCR amplification, and sequencing

Total genomic DNA was isolated from leaves samples using the FastDNA SPIN kits (MP Biomedicals, Santa Ana, CA, USA) according to the manufacturer’s instructions and stored at −20 °C until use. DNA concentration and purity were verified spectrophotometrically using the A260/280 ratio on a BioTek Synergy H1 Hybrid Multi-Mode Microplate Reader. PCR amplification was conducted in triplicate 50 μL reactions containing 2x Premix Taq (Takara Biotechnology, China), 1 μL of each primer (10 μM), and 3 μL of DNA template (20 ng/μl). Thermal cycling parameters included an initial denaturation at 94 °C for 3 min, followed by 30 cycles of denaturation at 94 °C for 30 s, annealing at 52 °C for 30 s, and extension at 72 °C for 30 s, with a final elongation at 72 °C for 10 min. Triplicate amplicons from each sample were pooled, confirmed by 2% agarose gel electrophoresis, and purified using the QIAamp DNA Micro Kit (Qiagen, USA). Purified amplicons were sequenced on an Illumina HiSeq 2,500 platform, generating 250 bp paired-end reads. The same PCR amplification protocol (primers, reagent concentrations, and thermal cycling parameters) was used for both the bacterial (16S rRNA) and fungal (ITS2) amplicons. The dataset and raw metagenomics generated during this research were deposited in the NCBI database, and the nucleotide sequences have been assigned GenBank accession numbers QB070827–QB073174. The data can be found online at: https://www.ncbi.nlm.nih.gov/nuccore/QB070827.

### 2.4 Bioinformatics and statistical analysis

Raw sequence reads were subjected to quality control and processing. Low-quality bases and adapter sequences were trimmed using Trimmomatic and Cutadapt (v1.9.1) (Nabakooza et al., 2024). High-quality paired-ends were then merged, quality filtered, and denoised to infer amplicon sequence variants (ASVs) using the UNOISE3 algorithm implemented in USEARCH (v11) (Edgar, 2016). Representative sequences were taxonomically annotated using the SILVA database (release 138) with the Ribosomal Database Project (RDP) classifier, and fungal (ITS2) sequences were classified against the UNITE database using the same approach (Yilmaz et al., 2014). Alpha-diversity metrics (Shannon, observed ASVs) were computed using the *vegan* package in R, while Beta-diversity was evaluated through Principal Coordinate Analysis (PCoA) based on Bray–Curtis dissimilarity matrices. Redundancy Analysis (RDA) and correlation-based network heatmaps were performed to identify chemical variables significantly associated with microbial community composition. Pearson correlations among the chemical variables were visualized as a heatmap combined with a correlation network using the R package linkET (Huang, 2021). The Shannon diversity index of each material was correlated with the measured chemical variables using Spearman’s rank correlation and visualized as a hub-and-spoke network with the R package ggClusterNet (Wen et al., 2022), with edge color indicating the direction of the correlation and edge width indicating its strength. All statistical analyses were performed in R, with significance set at *p* < 0.05.

## 3 Results

### 3.1 Changes in basic chemical constituents during fermentation

The chemical composition of both filler and wrapper cigar tobacco leaves exhibited marked temporal variations during fermentation (Table 1). In filler leaves, reducing and total sugars showed a continuous decline throughout fermentation, decreasing from 0.57 and 1.05% at the initial stage to 0.21 and 0.51% at 89 days, respectively. A similar downward trend was observed in wrapper leaves, where reducing sugars decreased from 0.59 to 0.20% and total sugars from 0.90 to 0.38%. This steady reduction reflects the progressive microbial utilization and enzymatic degradation of carbohydrates as energy sources during fermentation. Total nitrogen content in filler leaves initially increased from 3.26% to a peak of 3.60% at 18 days before declining to 3.02% by day 89, whereas wrapper leaves displayed a different pattern, peaking sharply at 4% on day 10 before decreasing to 2.61% by day 89. These contrasting patterns suggest stage-dependent shifts in nitrogen dynamics during fermentation, potentially reflecting differences in substrate turnover and microbial activity between filler and wrapper leaves. The potassium content generally increased during fermentation, with filler leaves showing a rise from 3.35 to 4.47% and wrapper leaves from 4.85 to 5.03% by the end of process. Conversely, chlorine exhibited minor fluctuations, with filler leaves ranging from 0.74 to 0.79% and wrapper leaves showing a modest increase from 0.50 to 0.62%. Total alkaloid content followed a similar trajectory, increasing in filler leaves during early fermentation, reaching a maximum of 4.94% at day 18, then decreasing to 3.66% by day 89. In contrast, alkaloid concentrations in wrapper leaves declined from 3.79 to 2.37%, suggesting differential degradation rates linked to leaf type and microbial activity.

**Table 1:** Chemical composition of cigar tobacco filler and wrapper leaves during fermentation.

T	RS %	TS %	TN %	K %	Cl %	TA %
F0	0.57 ± 0.018a	1.05 ± 0.002a	3.26 ± 0.01d	3.35 ± 0.007 g	0.74 ± 0.006e	4.12 ± 0.01c
F10	0.33 ± 0.009ef	0.56 ± 0.02e	3.58 ± 0.007b	4.58 ± 0.012c	0.76 ± 0.002 cd	4.61 ± 0.01b
F18	0.36 ± 0.009de	0.61 ± 0.001e	3.6 ± 0.007b	4.31 ± 0.007e	0.9 ± 0.004a	4.94 ± 0.04a
F49	0.29 ± 0.009f	0.58 ± 0.006e	3.44 ± 0.007c	4.04 ± 0.003f	0.77 ± 0.001bc	4.44 ± 0.006c
F89	0.21 ± 0.007 g	0.51 ± 0.01f	3.02 ± 0.014d	4.47 ± 0.016d	0.79 ± 0.004b	3.66 ± 0.01e
W0	0.59 ± 0.009a	0.9 ± 0.02b	3.18 ± 0.02d	4.85 ± 0.016b	0.5 ± 0.002 h	3.79 ± 0.01d
W10	0.41 ± 0.008c	0.8 ± 0.007b	4 ± 0.03a	3.18 ± 0.009 g	0.75 ± 0.01de	3.38 ± 0.03 g
W18	0.45 ± 0.007b	0.74 ± 0.01c	3.02 ± 0.007d	4.52 ± 0.02 cd	0.57 ± 0.001 g	3.54 ± 0.02f
W49	0.37 ± 0.003d	0.67 ± 0.01d	2.86 ± 0.014e	4.84 ± 0.012b	0.55 ± 0.005 h	2.82 ± 0.007 h
W89	0.2 ± 0.006 g	0.38 ± 0.007 g	2.61 ± 0.03e	5.03 ± 0.03a	0.62 ± 0.007f	2.37 ± 0.03i

T, Treatment; F, Filler leaves; W, Wrapper leaves; 0, 10, 18, 49, 89 = days of fermentation; RS, Reducing Sugars; TS, Total Sugars; TN, Total Nitrogen; K, Potassium; Cl, Chlorine; TA, Total Alkaloids. Different lowercase letters indicate significant differences among treatments at *p* < 0.05 using Tukey’s HSD test (*n* = 3).

### 3.2 Changes in volatile compound classes during fermentation

The major volatile compound classes of both filler and wrapper tobacco leaves exhibited distinct temporal dynamics during the fermentation process (Figure 1). Total acid content increased gradually in both leaf types, with a pronounced rise in wrapper leaves at 89 days, although variability was higher at this stage. Total alcohols fluctuated significantly over time, peaking in filler leaves at 10 days (*p* < 0.01) before decreasing, whereas wrapper leaves showed delayed accumulation at 18 days followed by stabilization. Total aldehydes increased sharply in filler leaves at 18 days (*p* < 0.05), while wrapper leaves maintained comparatively lower levels throughout most of the process, except for a modest increase at 89 days (*p* < 0.001). Total esters accumulated rapidly during early fermentation, reaching a maximum at 10–18 days in both leaf types, followed by a marked decline in later stages (*p* < 0.05). Total heterocyclic compounds remained relatively stable across fermentation, with only a transient increase at 18 days in both filler and wrapper leaves. In contrast, total ketones exhibited pronounced early peaks, particularly in filler leaves at 10 days (*p* < 0.05), indicating stronger early-stage shifts in volatile carbonyl formation during the initial phase of fermentation.

**Figure 1:**
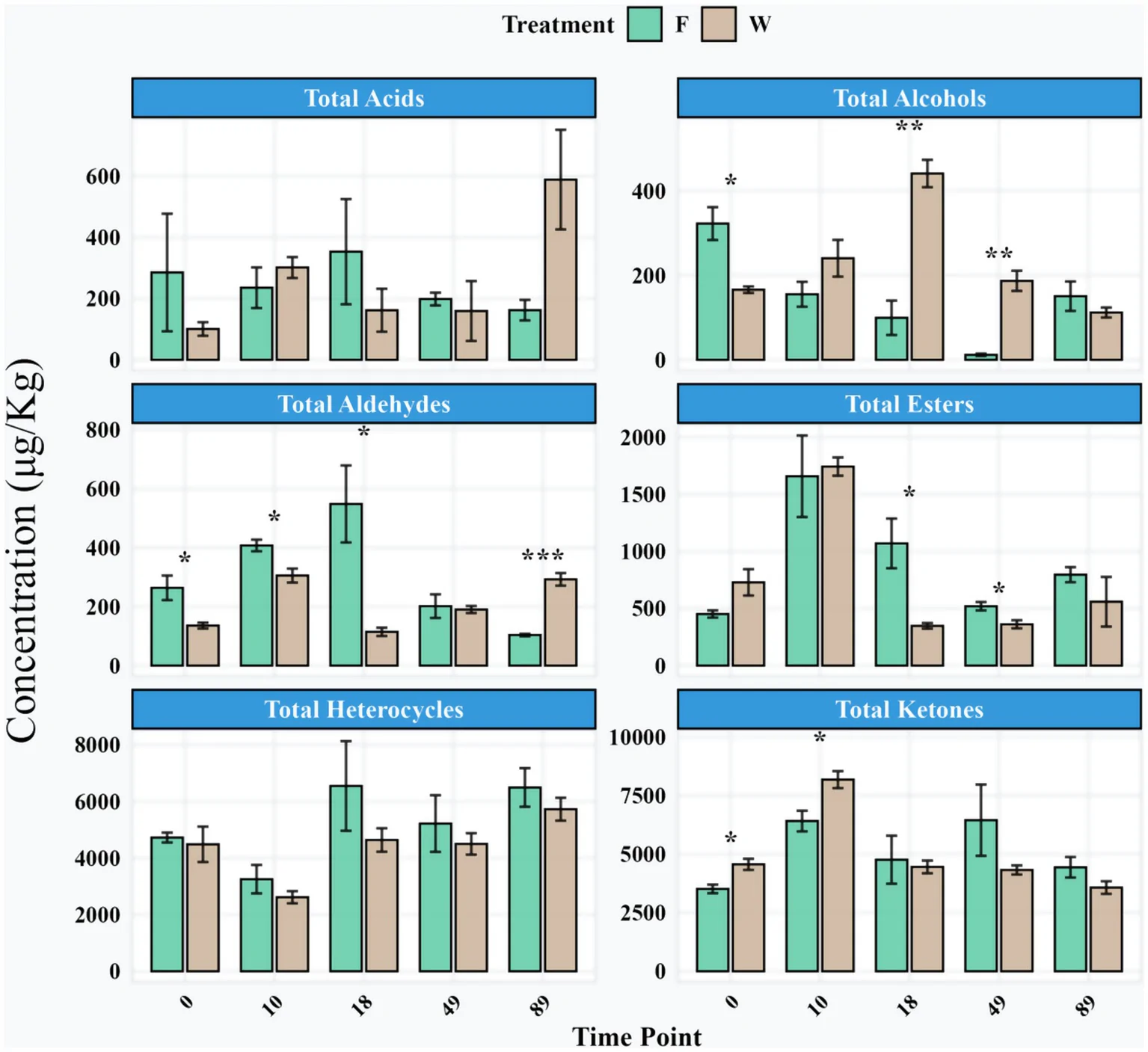
Bar graphs representing concentrations of volatile organic compounds across different treatments. Data are presented as mean ± SEM (error bars). Significant differences between treatments at each time point are indicated by asterisks (*p < 0.05, **p < 0.01, ***p < 0.001). F, Filler leaves; W, Wrapper leaves. 0, 10, 18, 49, 89 indicates different days of fermentations.

### 3.3 Microbial diversity and community composition during fermentation

The bacterial and fungal communities of both filler and wrapper leaves showed pronounced temporal dynamics during fermentation (Figure 2). Venn diagrams revealed stage-dependent variation in bacterial amplicon sequence variants (ASVs) richness, with filler leaves exhibiting higher total bacterial richness than wrapper leaves (Figure 2A). Unique bacterial ASVs were most abundant at day 18 in filler leaves (8.43%), and at day 10 in wrapper leaves (7.28%), suggesting distinct bacterial assembly trajectories between the two materials. In contrast, fungal ASVs (Figure 2B) were less diverse overall, and both materials displayed an increase in shared taxa over time, suggesting community stabilization toward later stages. The alpha diversity indices further confirmed these trends. Bacterial Shannon diversity (Figure 2C) declined progressively during fermentation, particularly after day 18, with a steeper reduction in wrapper leaves, indicating a transition from an early diverse community to a more specialized late-stage assemblage. Fungal diversity (Figure 2D), however, exhibited a transient rise in mid-fermentation (day 18–49), followed by a reduction at day 89, suggesting temporal shifts in dominant fungal populations related to substrate depletion and environmental stabilization. At the phylum level, Proteobacteria dominated bacterial communities throughout the fermentation (Figure 2E; Supplementary Table S1), particularly during the initial stages (10–18 days). However, the relative abundance of Firmicutes increased markedly in later stages, especially in filler leaves, implying a succession towards heat and stress tolerant species. Minor phyla including Actinobacteria, Bacteroidetes, and Cyanobacteria occurred sporadically with low abundance. For Fungi, Ascomycota consistently represented the most dominant phylum (Figure 2F; Supplementary Table S2), followed by Basidiomycota, which become more prevalent during the middle and late fermentation stages. Members of Mortierellomycota and unclassified fungi appeared occasionally, indicating a complex but transient fungal population.

**Figure 2:**
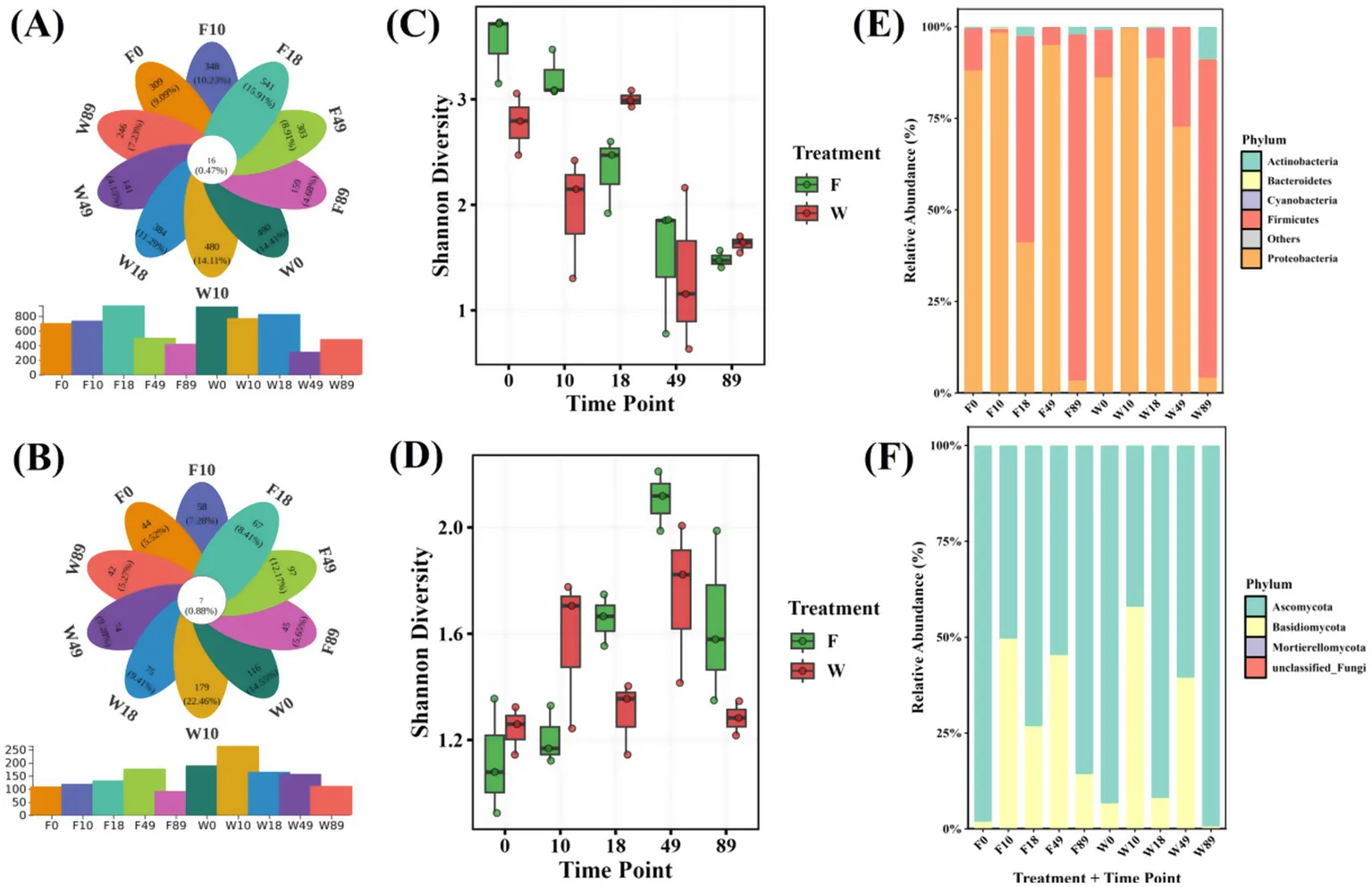
Microbial community composition and diversity across different treatments. Venn diagrams showing the number of unique and shared amplicon sequence variants (ASV) between bacteria **(A)** and fungi **(B)**. Boxplots of the shannon diversity index illustrating alpha diversity within bacterial **(C)** and fungal **(D)** communities. Stacked bar charts indicate relative abundance of the dominant bacterial **(E)** and fungal **(F)** phyla (> 1% abundance in at least one sample). F, Filler leaves; W, Wrapper leaves. 0–10–18-49-89 indicate different time point (days).

At bacterial genus level (Figure 3A; Supplementary Table S3), *Acinetobacter*, *Staphylococcus*, and *Kosakonia* dominated across both treatments, although their temporal abundance varied markedly. In filler leaves, *Acinetobacter* increased from day 10 onward, reaching maximum abundance at day 49 before declining by day 89. In contrast, wrapper leaves were initially colonized by *Pseudomonas* and *Ralstonia*, which were gradually replaced by *Acinetobacter* and *Staphylococcus* in the later stages, suggesting a shift from early oxidative degraders to stress-tolerant fermentative taxa. For fungi (Figure 3B; Supplementary Table S4), *Aspergillus* was the predominant genus across all time points, with co-occurrence of *Papiliotrema*, *Eutypella*, and *Penicillium* depending on fermentation stage. Filler leaves displayed early dominance of *Aspergillus* and *Papiliotrema*, whereas wrapper leaves exhibited a delayed rise in *Aspergillus* accompanied by *Eutypella* and *Neocucurbitaria*, indicating treatment specific fungal colonization. Figures 3C,D shows the top three abundant ASVs in bacteria and fungi. *Acinetobacter* (ASV_15786 and ASV_19787) showed a progressive increase, peaking around day 49 in both treatments but with significantly higher relative abundance in wrapper leaves. *Staphylococcus* (ASV_12333) was detected primarily in the late fermentation phase, coinciding with reduced sugar content and stable alkaloid levels (Figure 3C; Supplementary Table S5). Among fungi, *Aspergillus* ASVs (ASV_2859 and ASV_3119) dominated throughout fermentation, with markedly higher abundance in filler leaves, while *Papiliotrema* (ASV_1402) increased gradually, reaching its highest abundance by day 89 in wrapper leaves (Figure 3D; Supplementary Table S6).

**Figure 3:**
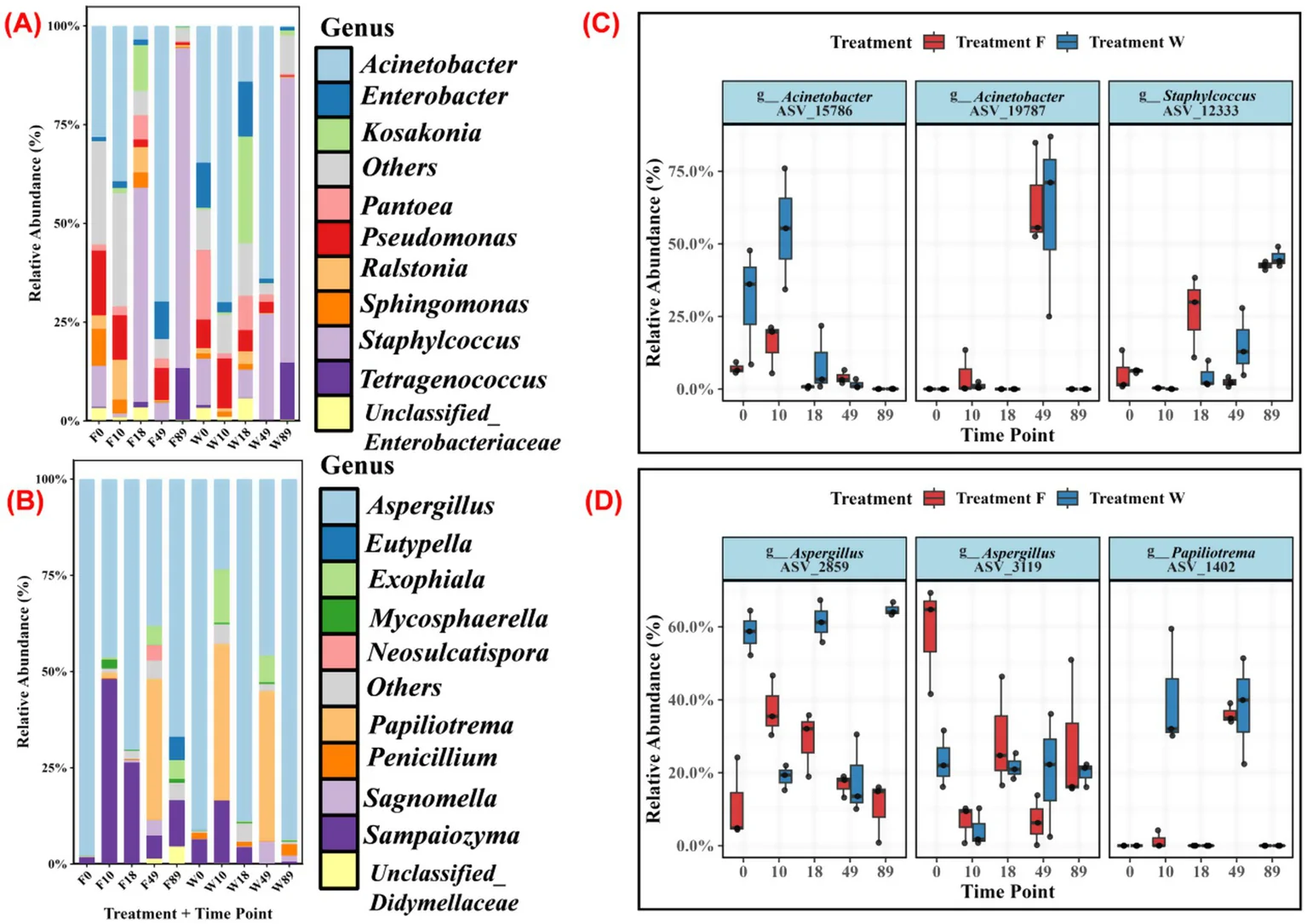
Microbial community composition and diversity across different treatments. Stacked bar charts indicate relative abundance of the dominant bacterial **(A)** and fungal **(B)** genera (> 1% abundance in at least one sample). The relative abundance of top 3 ASVs among bacterial **(C)** and fungal **(D)** communities. F = Filler leaves; W = Wrapper leaves. 0-10-18-49-89 indicate different time point (days).

### 3.4 Beta diversity and temporal succession of microbial communities

Principal coordinate analysis (PCoA) based on Bray–Curtis dissimilarity revealed clear treatment-related shifts in microbial community structure during fermentation (Figure 4). For bacterial communities, the first two coordinates explained 55.2% of the total variation (PCo1; 33.38%; PCo2; 21.8%), with partial separation between filler and wrapper leaves (Figure 4A). PERMANOVA confirmed that fermentation time explained a substantial proportion of bacterial community variation (R^2^ = 0.5735, *p* = 0.001), whereas treatment had a smaller effect (R^2^ = 0.0361, *p* = 0.35). Sample positions along the ordinates suggested a directional temporal shift from early to late-stage bacterial assemblages. For fungal communities, PCoA also displayed clear temporal differentiation, accounting for 61% of total variance (PCo1: 36.5%; PCo2: 24.45%). Similar to bacteria, fermentation time (R^2^ = 0.4154, *p* = 0.001) had a significant effect on community composition, whereas treatment exerted a weaker but detectable influence (R^2^ = 0.078, *p* = 0.03). Early fermentation stages (0–10 days) were dominated by diverse fungal taxa, which became progressively simplified and converged by day 49 and 89, suggesting a process of community stabilization under selective environmental conditions within the fermentation pile (Figure 4B).

**Figure 4:**
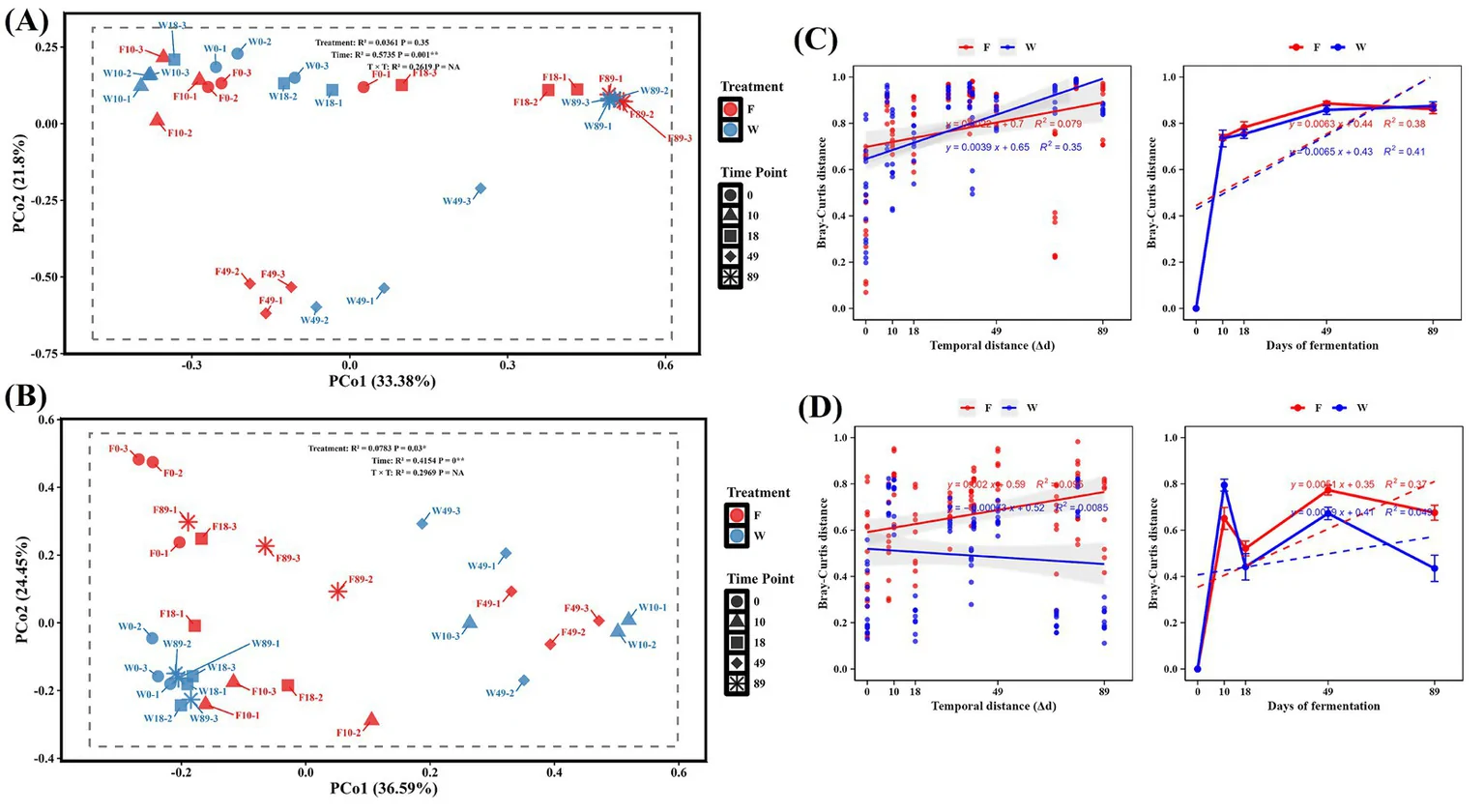
Beta diversity analysis and temporal turnover of microbial communities during fermentation across different time-points. **(A)** PCoA of bacterial communities based on Bray–Curtis dissimilarity. PERMANOVA results indicate that time (R^2^ = 0.5735, *p* = 0.001) was a significant driver of community variation, while treatment (R^2^ = 0.0361, *p* = 0.35) was non-significant. PCo1 and PCo2 explain 33.38 and 21.8% of the total variance, respectively. **(B)** PCoA of fungal communities based on Bray–Curtis dissimilarity. PERMANOVA results indicate that Time (R^2^ = 0.4154, *p* = 0.001) and Treatment (R^2^ = 0.0783, *p* = 0.03) was significant drivers of community variation. **(C)** Linear regression of Bray–Curtis dissimilarity, time, and bacterial community expressed as temporal distance (lag) and days of fermentation. Regression equations and R^2^ values are displayed for each plot. The W treatment exhibited a faster and more time-linear succession pattern than the F treatment. **(D)** Linear regression of Bray–Curtis dissimilarity, time, and fungal community expressed as temporal distance (lag) and days of fermentation. Regression equations and R^2^ values are displayed for each plot. The F treatment exhibited a faster and more time-linear succession pattern than the W treatment. F, Filler leaves; W, Wrapper leaves. 0-10-18-49-89 indicates different time point (days).

Bray-Curtis temporal distance analyses (Figures 4C,D) further confirmed directional microbial succession. In both bacterial and fungal communities, dissimilarity increased with fermentation duration, indicating progressive temporal turnover. For bacteria, the rate of change demonstrated a faster community turnover in wrapper leaves, suggesting a more dynamic microbial adaptation (Figure 4C). In contrast, fungal communities displayed slower but consistent compositional changes, with community dissimilarity stabilizing after day 49, indicating the establishment of a stable late stage fungal assemblage (Figure 4D).

### 3.5 Relationships between microbial communities and chemical composition during fermentation

Correlation heatmaps (Figures 5A,B) revealed distinct associations between dominant microbial taxa and chemical components of cigar tobacco during fermentation. Among bacterial phyla, Firmicutes showed significant positive correlation with total heterocycles and negative significant correlation with total ketones, whereas Proteobacteria showed the opposite trend (Figure 5A; Supplementary Table S7). Proteobacteria also showed positive correlation with reducing sugars, total nitrogen, and total alcohols, and negative correlation with potassium content. Bacteroidetes showed positive significant correlation with total esters and total acids, but negative correlation with total sugars and total alcohols. These patterns indicate that bacterial community composition covaried with changes in carbohydrates, nitrogen, and volatile profiles during fermentation, but they do not demonstrate direct metabolic activity by specific taxa. Among fungal genera, *Aspergillus* showed negative correlation with total nitrogen, chlorine, total esters and negative significantly correlated with total ketones (Figure 5B). *Sampaiozyma* showed positive significant correlation with total alkaloids, total nitrogen, total esters, and total ketones, while negative correlated with reducing sugars, potassium and total alcohols. *Penicillium* was positive and significantly correlated with potassium. Overall, most fungal genera showed negative correlations with total sugars and total alkaloids (Figure 5B; Supplementary Table S8). These pattern suggest that shifts in fungal community composition were associated with aromatic volatiles during mid- to late-stage fermentation.

**Figure 5:**
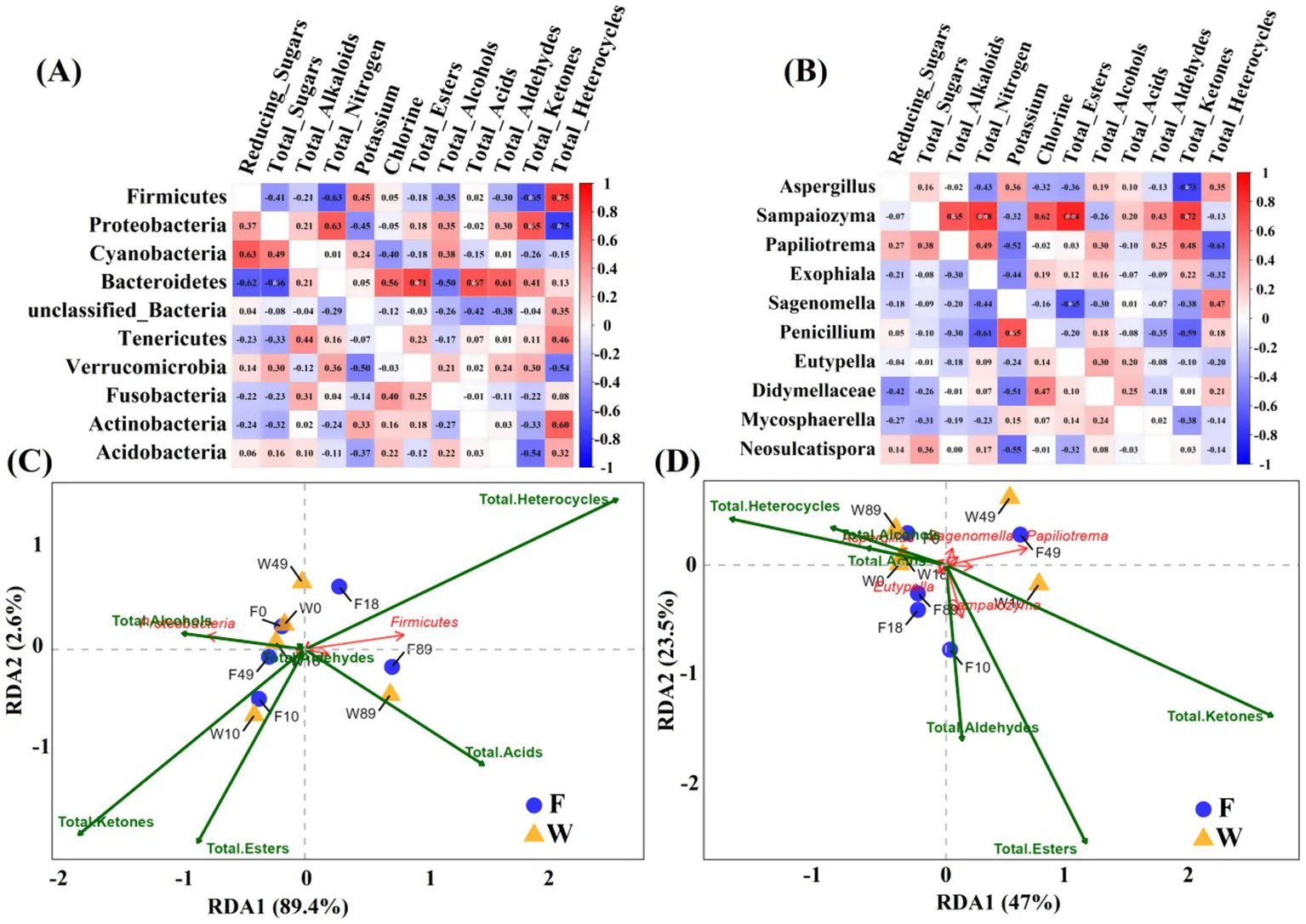
Correlation heatmaps and redundancy analysis. **(A)** Spearmen correlation heatmap between dominant bacterial phyla and environmental factors. **(B)** Spearmen correlation heatmap between dominant fungal genera and environmental factors. Red color indicate positive correlations and blue color indicate negative correlations. Only significant correlations (*p* < 0.05) are displayed. **(C)** Redundancy analysis between dominant bacterial phyla and volatile organic compounds, **(D)** Redundancy analysis between dominant fungal genera and volatile organic compounds. Panels **(C,D)** display one point per treatment × time-point combination, each summarizing *n* = 3 replicates. F, Filler leaves; W, Wrapper leaves. 0-10-18-49-89 indicates different time point (days).

Redundancy analysis (RDA) further summarized the covariation between microbial community composition and chemical transformation (Figures 5C,D). For bacterial communities, the first two RDA axes explained 89.4 and 2.6% of the constrained variation. Firmicutes aligned strongly with total aldehydes, whereas late stage samples (F89, W89) were positioned closer to total acids and heterocycles, indicating that these chemical traits co-varied with late fermentation bacterial assemblages (Figure 5C). For fungi, the first two RDA axes explained 47 and 23.5% of the total variation (Figure 5D). *Aspergillus* aligned with heterocycles and alcohols, whereas *Papiliotrema*, *Sampaiozyma*, and *Eutypella* were positively associated with total esters, aldehydes, and ketones. These ordination patterns identify statistically supported associations between dominant fungal genera and volatile classes; however functional verification would require targeted metabolomics, culture based assays, or metagenomic approaches. Moreover, filler and wrapper leaves exhibited partially distinct clusters indicating that leaf type influenced the coupled trajectory of microbial population and chemical evolution during fermentation.

### 3.6 Network relationships between microbial diversity and chemical components dynamics

The Correlation-based network analysis revealed significant associations between microbial community structure and chemical dynamics across fermentation stages (Figure 6; Supplementary Table S9). In the bacterial network, both positive and negative correlations linked bacterial diversity with chemical traits across fermentation stages in filler and wrapper leaves (Figure 6A), reflecting coordinated but correlative shifts during fermentation. Positive correlations were particularly evident among total sugars, reducing sugars, and total alkaloids. In contrast, many negative links connected with secondary chemical traits such as acids and aldehydes, consistent with progressive substrate depletion and product accumulation. Detailed correlations and *p* values are provided in Supplementary Table S10. For fungi, a denser and more interconnected network was observed (Figure 6B), indicating that fungal community change tracked a broad range of chemical variables. Positive correlations were observed among reducing sugars, total sugars, total alkaloids, total nitrogen, while negative correlations were linked with potassium, chlorine and total heterocycles (Figure 6B). Detailed correlation and *p* values are provided in Supplementary Table S11.

**Figure 6:**
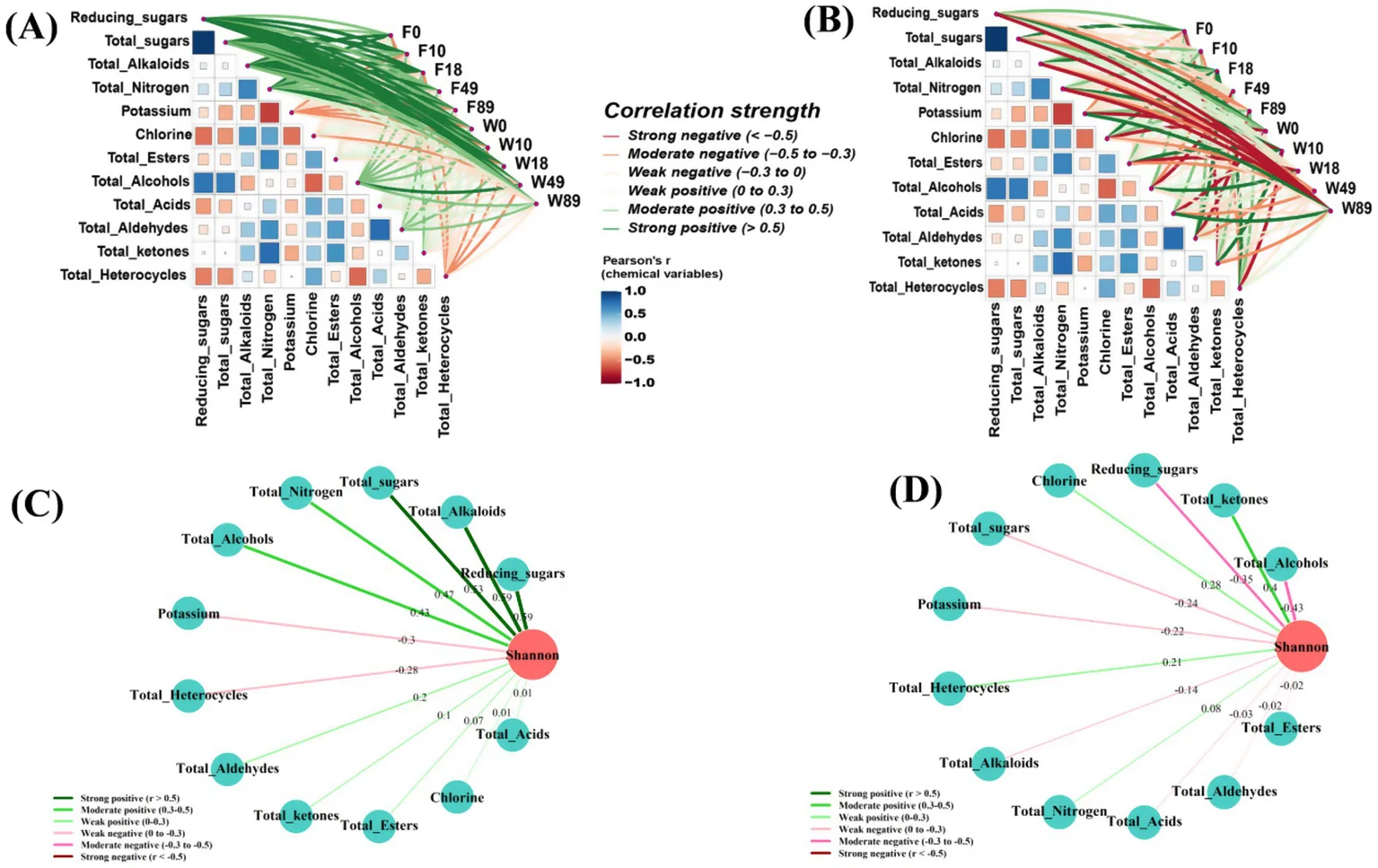
Heatmap-network and correlation analysis between microbial communities and chemical variables. **(A)** Heatmap-network analysis showing significant correlations between bacterial communities and key chemical components. **(B)** Heatmap-network analysis showing significant correlations between fungal communities and key chemical components. Curved lines radiate from each fermentation timepoint (F0–F89, filler; W0–W89, wrapper) to the chemical variables they are significantly/notably correlated with; line color indicates correlation strength and direction (strong positive, *r* > 0.5; moderate positive, 0.3–0.5; weak positive, 0–0.3; weak negative, −0.3–0; moderate negative, −0.5 to −0.3; strong negative, r < −0.5), following the legend shown between panels **(A,B)**. **(C,D)** Hub-and-spoke correlation networks showing Spearman’s rank correlations between the Shannon diversity index (central red node) and each chemical variable (teal nodes) for filler **(C)** and wrapper **(D)** leaves across all fermentation timepoints. Edge color and width indicate correlation strength and direction. F, Filler leaves; W, Wrapper leaves. 0-10-18-49-89 indicates different time point (days).

Further correlations analysis was performed using the overall bacterial and fungal Shannon diversity (Figures 6C,D). For bacterial communities, Shannon diversity was positively correlated with reducing sugars (*r* = 0.59), total alkaloids (*r* = 0.59), and total sugars (*r* = 0.53), total nitrogen (*r* = 0.47), and total alcohols (*r* = 0.43). This pattern suggests that higher bacterial diversity coincided with the earlier, substrate richer phases of fermentation. Conversely, bacterial diversity showed weak to moderate negative correlations with potassium and total heterocycles (Figure 6C). In contrast, fungal diversity showed moderate positive correlations with total ketones (*r* = 0.43), and weak positive correlations with chlorine, total heterocycles and total nitrogen (Figure 6D), whereas negative correlations with sugars. These trends indicate that fungal diversity shifted as the chemical environment changed during fermentation; however, they should be interpreted as ecological associations rather than direct evidence that microbial diversity itself drove chemical conversion.

## 4 Discussion

This study provides a comparative view of microbial and chemical changes in cigar filler and wrapper leaves during bulk fermentation. Key observations included: (i) progressive depletion of reducing and total sugars across the 89-day fermentation process; (ii) distinct temporal patterns of total alkaloids, nitrogen, and inorganic constituents between filler and wrapper leaves; (iii) a shift from relatively diverse early microbial communities to late-stage communities enriched in *Acinetobacter*, *Staphylococcus*, *Aspergillus*, and *Papiliotrema*; (iv) statistically significant correlations between microbial community variation and multiple chemical parameters; and (v) leaf-type-specific differences in both microbial and chemical dynamics. Below, these findings are interpreted in relation to previous tobacco fermentation studies while taking into account the correlative nature of the community-chemistry analyses.

### 4.1 Carbohydrate and alkaloid degradation, microbial implications

The decline in reducing sugars and total sugars in both leaf types (from 0.57 to 0.21% for filler, 0.59 to 0.20% for wrapper) over the fermentation period is consistent with substantial microbial and enzymatic consumption of carbohydrate substrates. This aligns with prior reports identifying carbohydrate depletion as a marker of tobacco fermentation and sugars as a major carbon and energy sources for microbial metabolism (Liu et al., 2021). In contrast, total nitrogen and alkaloids showed non-monotonic trajectories. The transient increase in total nitrogen around day 10–18, likely reflects concentration effects from dry-matter loss, redistribution among nitrogen pools, and temporary microbial biomass accumulation, rather than net nitrogen generation (Grzyb et al., 2021). The different alkaloid trajectories between filler and wrapper leaves may similarly reflect differences in initial substrate accessibility and tissue composition. In filler leaves, the early rise in total alkaloids may have resulted from concentration effects or release of bound or compartmentalized alkaloid pools before later decline, whereas wrapper leaves showed a more direct decrease from beginning. This pattern may reflect differences in tissue composition, substrate accessibility, or the timing of nitrogenous compounds transformation between filler and wrapper leaves (Wang et al., 2024). Wrapper leaves may have fewer residual alkaloids and thus proceeded more directly to breakdown. Previous work in flue-cured tobacco found that microbial succession leads to alkaloid versus derivative transformations, including benzene-ring compounds, during aging (Wang et al., 2025). These patterns are consistent with fermentation stage dependent transformation of nitrogenous compounds, but the present data do not identify the responsible enzymes or taxa. Therefore, associations between late-stage genera such as *Staphylococcus* and *Acinetobacter* with alkaloid decline or volatile reconfiguration should be interpreted as correlative rather than causal.

### 4.2 Microbial succession and linking to functionality

The succession pattern observed in this study, an initial phase of higher diversity followed by reduced diversity and dominance of fewer taxa, is broadly consistent with other tobacco fermentation studies (Zhang L. Y. et al., 2024). The progressive decline in bacterial Shannon diversity, especially after day 18, likely reflects the combined effects of multiple deterministic selection pressure rather than any single factor (Zhang et al., 2025). As fermentation proceeds, self-heating, repeated turning, moisture redistribution, oxygen gradients, and depletion of readily metabolizable substrates narrow ecological niches, while competitive dominance by better adapted genera becomes more pronounced. The replacement of early, abundant genera such as *Pseudomonas* and *Ralstonia* by *Acinetobacter* and *Staphylococcus* is therefore consistent with increased tolerance of later-stage pile conditions and a shift in the available substrate spectrum, although taxon specific physiological validation is still needed (Tao et al., 2024). Leaf type appeared to exert a weaker effect on bacterial communities than fermentation time, but a stronger effect on fungal communities. Consistent with this pattern, fermentation stage has been shown to explain more variation in cigar-leaf microbial diversity than growing location or leaf source, and substrate composition more strongly shapes fungal than bacterial community structure in other systems (Habtewold et al., 2020; Zhang et al., 2023). These differences may indicate that bacterial communities were governed mainly by the convergent fermentation environment shared by both piles, whereas fungal communities remained more sensitive to substrate structure, surface properties, and leaf-specific chemistry. From a processing perspective, filler and wrapper leaves likely provide different ecological templates: filler leaves are generally more heterogeneous in tissue composition, whereas wrapper leaves are typically thinner and more structurally uniform. Such contrasts could influence moisture retention, oxygen diffusion, and substrate accessibility leaf structural and chemical features already shown to predict phyllosphere bacterial colonization in other systems (Yadav et al., 2005) thereby altering the tempo of microbial succession. However, because the two leaves were from different cultivars, this interpretation should be viewed as a process-level comparison of commercial filler and wrapper leaves rather than as a genotype-controlled test of intrinsic leaf-type effects.

### 4.3 Microbial-chemical coupling during flavor-associated transformation

Correlation and ordination analyses showed that microbial community variation co-occurred with systematic chemical change during fermentation. Bacterial diversity was positively associated with reducing sugars, total nitrogen and total alkaloids, whereas later stage communities were associated with acids, aldehydes, ketones, or heterocycles, supporting a staged transition from substrate-rich to chemically transformed states. This pattern agrees with general ecological succession theory and with previous tobacco studies linking fermentation stage to coordinated changes in microbial communities and flavor related compounds (Jia et al., 2025; Wang et al., 2025). The coupling of microbial turnover with production of volatile compounds (esters, ketones, aldehydes) is congruent with previous studies showing that core bacterial genera such as *bacillus* and *Pseudomonas* are significantly correlated with flavor compounds such as benzaldehyde or terpenoids in tobacco fermentation (Huang et al., 2022). However, the present analyses do not demonstrate that particular taxa produced specific compounds. Some chemical classes, especially heterocyclic compounds, remained comparatively stable despite pronounced community turnover, suggesting that their abundance may depend more on precursor limitation, non-microbial thermochemical reactions, or later stage equilibration than on direct shifts in microbial abundance at the taxonomic resolution used here. Thus, the principal contribution of this data set is not direct functional proof, but a comparative map of which microbial transitions and chemical trajectories co-vary during filler and wrapper fermentation.

### 4.4 Limitations and future directions

While this study integrates comprehensive chemical profiling and amplicon sequencing across fermentation time, several limitations should be acknowledged. First, although filler and wrapper leaves were fermented under identical processing conditions, varietal background remains a confounding factor and may contribute to differences in initial chemistry and microbial selection. Future work using the same cultivar for both leaf types would help isolate leaf-type effects per se. Second, samples were collected from a standardized core location within each pile, which improves temporal comparability but does not resolve full within-pile spatial heterogeneity. Third, each material was fermented as a single 50 kg pile, and the three replicates analyzed at each time point were sub-samples from that one pile rather than independent pile-level replicates; consequently, the Tukey’s HSD, PERMANOVA, and related significance tests reported here characterize within-pile variability and should not be interpreted as biological replication at the pile level. Confirming these patterns would require repeating the fermentation in multiple independent piles per material. Fourth, the study ends at 89 days although industrial cigar-leaf fermentation can proceed for months. Longer-term successional phases may reveal further community shifts and flavor maturation.

## 5 Conclusion

In sum, cigar leaf fermentation is a time-structured process in which microbial succession and chemical transformation are tightly coupled. Under a shared fermentation environment, filler and wrapper leaves exhibited distinct temporal trajectories in sugars, alkaloids, volatiles, and microbial composition, indicating that leaf type influences fermentation outcomes. Correlation, redundancy, and network analysis identified robust associations between community variation and biochemical change, but these results should be interpreted as ecological relationships rather than direct proof of causality. By clarifying comparative fermentation trajectories in two commercially important cigar leaves, this study provides a useful basis for process monitoring, hypothesis generation, and future optimization of tobacco and other plant-based fermentation systems.

## Data Availability

The dataset and raw metagenomics generated during this research were deposited in the NCBI database, and the nucleotide sequences have been assigned GenBank accession numbers QB070827–QB073174. The data can be found online at: https://www.ncbi.nlm.nih.gov/nuccore/QB070827.
