# Supplementary material for: Comparative analysis of microbial succession and biochemical changes in cigar tobacco filler and wrapper leaves during fermentation
Source: Front Microbiol. 2026 Oct 2;17:1922631. doi: 10.3389/fmicb.2026.1922631 (PMC13671968; doi:10.3389/fmicb.2026.1922631)
Supplement: Supplementary file 1 [file Data_Sheet_1.DOCX]

**Comparative Analysis of Microbial Succession and Biochemical Changes in Filler and Wrapper Leaves of Cigar Tobacco During Fermentation**

Zhaoliang Geng^1†^, Bin Cai^1†^, Yanqi Hu^2^, Beisen Lin^1^, Tongjing Yan^1^, Huajun Gao^1*^, Abdullah Khan^3*^, Xinghua Ma^3*^

^1^ Hainan Provincial Branch of China National Tobacco Corporation, Haikou 571100, China

^2^ China Tobacco Shandong Industrial Co., Ltd., Jinan 250100, China

^3^ Key Laboratory of Tobacco Biology and Processing, Ministry of Agriculture and Rural Affairs, Tobacco Research Institute of Chinese Academy of Agricultural Sciences, Qingdao 266101, China

^†^These authors contributed equally to this work

zhaolianggeng1104@126.com

[caibin_hcri@qq.com](mailto:caibin_hcri@qq.com)

[*gaohuajun_81@163.com](mailto:*gaohuajun_81@163.com)

*abdullahgxu@gmail.com

*maxinghua@caas.cn

Figure S1. Temperature and humidity dynamics during 90-day bulk fermentation of wrapper and filler leaves.

Table S1: Relative abundance of major bacterial phyla during fermentation across different treatments.

| T | F0 | F10 | F18 | F49 | F89 | W0 | W10 | W18 | W49 | W89 |
| --- | --- | --- | --- | --- | --- | --- | --- | --- | --- | --- |
| Firmicutes | 11.5% ± 7.8% | 1.1% ± 0.2% | 56.3% ± 18.1% | 4.8% ± 1.8% | 94.4% ± 1.0% | 12.9% ± 0.5% | 0.3% ± 0.1% | 8.0% ± 4.0% | 27.2% ± 13.7% | 86.9% ± 3.6% |
| Proteobacteria | 88.1% ± 7.8% | 98.3% ± 0.3% | 41.2% ± 18.1% | 95.0% ± 1.8% | 3.5% ± 0.9% | 86.3% ± 0.6% | 99.5% ± 0.2% | 91.5% ± 4.1% | 72.7% ± 13.7% | 4.2% ± 1.7% |
| Cyanobacteria | 0.1% ± 0.0% | 0.0% ± 0.0% | 0.0% ± 0.0% | 0.0% ± 0.0% | 0.0% ± 0.0% | 0.1% ± 0.0% | 0.0% ± 0.0% | 0.0% ± 0.0% | 0.0% ± 0.0% | 0.0% ± 0.0% |
| Bacteroidetes | 0.0% ± 0.0% | 0.3% ± 0.1% | 0.1% ± 0.1% | 0.0% ± 0.0% | 0.0% ± 0.0% | 0.0% ± 0.0% | 0.1% ± 0.0% | 0.0% ± 0.0% | 0.0% ± 0.0% | 0.1% ± 0.0% |
| unclassified_Bacteria | 0.0% ± 0.0% | 0.0% ± 0.0% | 0.0% ± 0.0% | 0.0% ± 0.0% | 0.0% ± 0.0% | 0.0% ± 0.0% | 0.0% ± 0.0% | 0.0% ± 0.0% | 0.0% ± 0.0% | 0.0% ± 0.0% |
| Tenericutes | 0.0% ± 0.0% | 0.0% ± 0.0% | 0.0% ± 0.0% | 0.0% ± 0.0% | 0.0% ± 0.0% | 0.0% ± 0.0% | 0.0% ± 0.0% | 0.0% ± 0.0% | 0.0% ± 0.0% | 0.0% ± 0.0% |
| Verrucomicrobia | 0.0% ± 0.0% | 0.0% ± 0.0% | 0.0% ± 0.0% | 0.0% ± 0.0% | 0.0% ± 0.0% | 0.0% ± 0.0% | 0.0% ± 0.0% | 0.0% ± 0.0% | 0.0% ± 0.0% | 0.0% ± 0.0% |
| Fusobacteria | 0.0% ± 0.0% | 0.0% ± 0.0% | 0.0% ± 0.0% | 0.0% ± 0.0% | 0.0% ± 0.0% | 0.0% ± 0.0% | 0.0% ± 0.0% | 0.0% ± 0.0% | 0.0% ± 0.0% | 0.0% ± 0.0% |
| Actinobacteria | 0.4% ± 0.2% | 0.3% ± 0.1% | 2.4% ± 0.7% | 0.1% ± 0.1% | 2.1% ± 1.1% | 0.7% ± 0.2% | 0.2% ± 0.1% | 0.4% ± 0.2% | 0.1% ± 0.1% | 8.8% ± 4.3% |
| Acidobacteria | 0.0% ± 0.0% | 0.0% ± 0.0% | 0.0% ± 0.0% | 0.0% ± 0.0% | 0.0% ± 0.0% | 0.0% ± 0.0% | 0.0% ± 0.0% | 0.0% ± 0.0% | 0.0% ± 0.0% | 0.0% ± 0.0% |

T = Treatment; F = Filler leaves; W = Wrapper leaves. 0-10-18-49-89 indicates different time point (days).

Table S2: Relative abundance of major fungal phyla during fermentation across different treatments.

| T | Ascomycota | Basidiomycota | unclassified_Fungi | Mucoromycota | unidentified | Mortierellomycota |
| --- | --- | --- | --- | --- | --- | --- |
| F0 | 98.01 ± 0.65 | 1.98 ± 0.65 | 0.01 ± 0.01 | 0.00 ± 0.00 | 0.00 ± 0.00 | 0.00 ± 0.00 |
| F10 | 49.79 ± 6.83 | 50.12 ± 6.83 | 0.01 ± 0.01 | 0.00 ± 0.00 | 0.01 ± 0.01 | 0.00 ± 0.00 |
| F18 | 74.31 ± 3.67 | 25.51 ± 3.67 | 0.14 ± 0.12 | 0.01 ± 0.01 | 0.02 ± 0.02 | 0.00 ± 0.00 |
| F49 | 54.56 ± 2.99 | 43.96 ± 4.14 | 1.44 ± 1.40 | 0.00 ± 0.00 | 0.03 ± 0.01 | 0.01 ± 0.00 |
| F89 | 85.49 ± 6.79 | 14.08 ± 6.93 | 0.02 ± 0.01 | 0.40 ± 0.40 | 0.01 ± 0.01 | 0.00 ± 0.00 |
| W0 | 93.03 ± 2.03 | 6.92 ± 2.09 | 0.03 ± 0.02 | 0.00 ± 0.00 | 0.01 ± 0.01 | 0.00 ± 0.00 |
| W10 | 42.00 ± 1.49 | 57.79 ± 1.37 | 0.14 ± 0.09 | 0.00 ± 0.00 | 0.05 ± 0.04 | 0.01 ± 0.00 |
| W18 | 91.66 ± 2.15 | 7.95 ± 2.12 | 0.38 ± 0.37 | 0.00 ± 0.00 | 0.00 ± 0.00 | 0.01 ± 0.00 |
| W49 | 61.20 ± 8.41 | 38.76 ± 8.41 | 0.00 ± 0.00 | 0.00 ± 0.00 | 0.02 ± 0.01 | 0.00 ± 0.00 |
| W89 | 98.68 ± 0.50 | 0.93 ± 0.28 | 0.39 ± 0.34 | 0.00 ± 0.00 | 0.00 ± 0.00 | 0.00 ± 0.00 |

T = Treatment; F = Filler leaves; W = Wrapper leaves. 0-10-18-49-89 indicates different time point (days).

Table S3: Relative abundance of major bacterial genera during fermentation across different treatments.

| T | Acinetobacter | Staphylococcus | Pseudomonas | Others | Ralstonia | Pantoea | Enterobacter | Sphingomonas | unclassified_Enterobacteriaceae | Kosakonia | Tetragenococcus |
| --- | --- | --- | --- | --- | --- | --- | --- | --- | --- | --- | --- |
| F0 | 28.17 ± 8.67 | 10.39 ± 7.48 | 16.42 ± 3.21 | 26.02 ± 2.38 | 3.43 ± 1.38 | 1.55 ± 0.68 | 1.01 ± 0.23 | 9.33 ± 5.78 | 3.28 ± 0.76 | 0.03 ± 0.01 | 0.31 ± 0.12 |
| F10 | 39.31 ± 3.94 | 0.07 ± 0.00 | 11.31 ± 2.35 | 28.66 ± 3.81 | 10.03 ± 4.49 | 2.24 ± 1.31 | 1.72 ± 0.42 | 3.53 ± 0.55 | 1.07 ± 0.15 | 1.29 ± 1.06 | 0.06 ± 0.05 |
| F18 | 3.45 ± 1.70 | 54.20 ± 17.43 | 1.88 ± 0.50 | 6.11 ± 1.19 | 6.46 ± 2.66 | 6.18 ± 5.69 | 1.43 ± 1.21 | 3.86 ± 1.63 | 3.37 ± 2.84 | 11.59 ± 11.29 | 1.42 ± 0.43 |
| F49 | 69.73 ± 9.01 | 4.58 ± 1.82 | 8.10 ± 2.59 | 4.90 ± 2.26 | 0.22 ± 0.08 | 2.43 ± 1.99 | 9.51 ± 5.02 | 0.40 ± 0.23 | 0.03 ± 0.03 | 0.00 ± 0.00 | 0.06 ± 0.01 |
| F89 | 0.23 ± 0.13 | 81.03 ± 1.47 | 0.68 ± 0.24 | 3.03 ± 0.91 | 0.45 ± 0.03 | 0.34 ± 0.24 | 0.10 ± 0.05 | 0.32 ± 0.12 | 0.32 ± 0.24 | 0.48 ± 0.21 | 13.00 ± 0.75 |
| W0 | 34.63 ± 13.81 | 11.69 ± 0.67 | 7.23 ± 1.43 | 10.21 ± 2.70 | 1.35 ± 0.38 | 17.63 ± 10.52 | 11.42 ± 6.21 | 1.34 ± 0.30 | 3.35 ± 1.49 | 0.50 ± 0.17 | 0.70 ± 0.21 |
| W10 | 69.95 ± 7.64 | 0.01 ± 0.00 | 12.60 ± 3.95 | 9.75 ± 3.90 | 0.83 ± 0.23 | 1.33 ± 0.49 | 2.57 ± 1.10 | 1.26 ± 0.61 | 0.96 ± 0.31 | 0.65 ± 0.23 | 0.00 ± 0.00 |
| W18 | 14.13 ± 5.66 | 7.07 ± 4.10 | 5.38 ± 2.50 | 13.32 ± 5.43 | 3.17 ± 0.95 | 8.68 ± 0.79 | 13.87 ± 8.36 | 1.45 ± 0.43 | 5.75 ± 2.49 | 27.03 ± 14.42 | 0.01 ± 0.01 |
| W49 | 63.96 ± 18.95 | 26.67 ± 14.76 | 2.76 ± 0.62 | 2.74 ± 1.10 | 0.16 ± 0.06 | 1.86 ± 1.26 | 1.19 ± 0.97 | 0.11 ± 0.06 | 0.00 ± 0.00 | 0.12 ± 0.09 | 0.02 ± 0.01 |
| W89 | 0.27 ± 0.07 | 72.09 ± 1.69 | 0.33 ± 0.08 | 9.70 ± 4.96 | 0.19 ± 0.03 | 0.44 ± 0.32 | 0.09 ± 0.07 | 0.08 ± 0.02 | 0.36 ± 0.22 | 1.21 ± 0.75 | 14.45 ± 3.11 |

T = Treatment; F = Filler leaves; W = Wrapper leaves. 0-10-18-49-89 indicates different time point (days).

Table S4: Relative abundance of major fungal genera during fermentation across different treatments.

| T | Aspergillus | Sampaiozyma | Papiliotrema | Exophiala | Sagenomella | Penicillium | Eutypella | Didymellaceae | Mycosphaerella | Neosulcatispora |
| --- | --- | --- | --- | --- | --- | --- | --- | --- | --- | --- |
| F0 | 97.61 ± 0.82 | 1.77 ± 0.65 | 0.35 ± 0.33 | 0.35 ± 0.35 | 0.35 ± 0.35 | 0.43 ± 0.30 | 0.35 ± 0.35 | 0.35 ± 0.35 | 0.41 ± 0.30 | 0.35 ± 0.35 |
| F10 | 45.42 ± 7.73 | 47.71 ± 5.24 | 1.36 ± 1.29 | 0.53 ± 0.30 | 0.00 ± 0.00 | 0.35 ± 0.05 | 0.15 ± 0.15 | 0.04 ± 0.03 | 2.40 ± 0.94 | 0.00 ± 0.00 |
| F18 | 68.05 ± 4.41 | 24.94 ± 3.71 | 0.01 ± 0.00 | 0.01 ± 0.00 | 0.59 ± 0.32 | 0.24 ± 0.14 | 0.00 ± 0.00 | 0.00 ± 0.00 | 0.23 ± 0.13 | 0.00 ± 0.00 |
| F49 | 36.81 ± 2.65 | 5.94 ± 3.06 | 35.04 ± 1.93 | 4.77 ± 1.30 | 3.83 ± 1.73 | 0.04 ± 0.02 | 0.00 ± 0.00 | 1.40 ± 1.36 | 0.07 ± 0.06 | 3.73 ± 2.20 |
| F89 | 65.10 ± 1.45 | 10.61 ± 6.79 | 0.00 ± 0.00 | 5.00 ± 4.96 | 0.06 ± 0.04 | 1.00 ± 0.98 | 5.99 ± 5.99 | 4.48 ± 4.46 | 0.84 ± 0.80 | 0.00 ± 0.00 |
| W0 | 90.62 ± 1.73 | 6.40 ± 2.06 | 0.00 ± 0.00 | 0.00 ± 0.00 | 0.08 ± 0.03 | 1.71 ± 0.48 | 0.01 ± 0.01 | 0.00 ± 0.00 | 0.16 ± 0.04 | 0.00 ± 0.00 |
| W10 | 22.07 ± 3.89 | 15.66 ± 6.92 | 38.61 ± 8.67 | 13.21 ± 3.12 | 0.01 ± 0.01 | 0.06 ± 0.01 | 0.45 ± 0.39 | 0.25 ± 0.17 | 0.40 ± 0.28 | 0.00 ± 0.00 |
| W18 | 86.88 ± 3.07 | 4.29 ± 2.85 | 0.03 ± 0.03 | 0.22 ± 0.21 | 0.25 ± 0.19 | 1.26 ± 0.11 | 0.00 ± 0.00 | 0.01 ± 0.00 | 0.39 ± 0.24 | 0.00 ± 0.00 |
| W49 | 44.75 ± 4.19 | 0.10 ± 0.10 | 37.77 ± 8.67 | 6.63 ± 2.78 | 5.55 ± 0.78 | 0.19 ± 0.16 | 0.00 ± 0.00 | 0.00 ± 0.00 | 0.56 ± 0.35 | 0.02 ± 0.02 |
| W89 | 92.69 ± 2.10 | 0.53 ± 0.16 | 0.00 ± 0.00 | 0.17 ± 0.11 | 1.63 ± 0.28 | 2.86 ± 0.97 | 0.07 ± 0.03 | 0.07 ± 0.07 | 0.42 ± 0.13 | 0.00 ± 0.00 |

T = Treatment; F = Filler leaves; W = Wrapper leaves. 0-10-18-49-89 indicates different time point (days).

Table S5: Abundance of top three bacterial ASV and their annotated genera.

| ASV_ID | Genus | Treatment | Time | Mean_Abundance | SD_Abundance |
| --- | --- | --- | --- | --- | --- |
| ASV_12333 | Staphylococcus | F | 0 | 3353 | 1485 |
| ASV_12333 | Staphylococcus | F | 10 | 213.33 | 56.22 |
| ASV_12333 | Staphylococcus | F | 18 | 17047 | 9066.85 |
| ASV_12333 | Staphylococcus | F | 49 | 1550 | 1102.31 |
| ASV_12333 | Staphylococcus | F | 89 | 27330.67 | 1574.28 |
| ASV_12333 | Staphylococcus | W | 0 | 4145 | 310.23 |
| ASV_12333 | Staphylococcus | W | 10 | 19 | 25.51 |
| ASV_12333 | Staphylococcus | W | 18 | 2666.67 | 2778.55 |
| ASV_12333 | Staphylococcus | W | 49 | 9466.33 | 6617.85 |
| ASV_12333 | Staphylococcus | W | 89 | 35745.33 | 9113.67 |
| ASV_15786 | Acinetobacter | F | 0 | 4593.33 | 1418.53 |
| ASV_15786 | Acinetobacter | F | 10 | 9459.33 | 5306 |
| ASV_15786 | Acinetobacter | F | 18 | 456.67 | 247.43 |
| ASV_15786 | Acinetobacter | F | 49 | 2586.67 | 1683.2 |
| ASV_15786 | Acinetobacter | F | 89 | 33 | 13.11 |
| ASV_15786 | Acinetobacter | W | 0 | 20934.33 | 13874.18 |
| ASV_15786 | Acinetobacter | W | 10 | 36045 | 15355.2 |
| ASV_15786 | Acinetobacter | W | 18 | 5306.67 | 7043.24 |
| ASV_15786 | Acinetobacter | W | 49 | 1007.33 | 896.91 |
| ASV_15786 | Acinetobacter | W | 89 | 60.67 | 35.57 |
| ASV_19787 | Acinetobacter | F | 0 | 28.67 | 33.84 |
| ASV_19787 | Acinetobacter | F | 10 | 2886 | 4785.79 |
| ASV_19787 | Acinetobacter | F | 18 | 0 | 0 |
| ASV_19787 | Acinetobacter | F | 49 | 40511.33 | 8473.19 |
| ASV_19787 | Acinetobacter | F | 89 | 0 | 0 |
| ASV_19787 | Acinetobacter | W | 0 | 0 | 0 |
| ASV_19787 | Acinetobacter | W | 10 | 689 | 656.54 |
| ASV_19787 | Acinetobacter | W | 18 | 0 | 0 |
| ASV_19787 | Acinetobacter | W | 49 | 40857 | 23466.96 |
| ASV_19787 | Acinetobacter | W | 89 | 0 | 0 |

T = Treatment; F = Filler leaves; W = Wrapper leaves. 0-10-18-49-89 indicates different time point (days).

Table S6: Abundance of top three fungal ASV and their annotated genera.

| ASV_ID | Genus | Treatment | Time | Mean_Abundance | SD_Abundance |
| --- | --- | --- | --- | --- | --- |
| ASV_1402 | Papiliotrema | F | 0 | 0 | 0 |
| ASV_1402 | Papiliotrema | F | 10 | 876.67 | 1514.97 |
| ASV_1402 | Papiliotrema | F | 18 | 7 | 2.65 |
| ASV_1402 | Papiliotrema | F | 49 | 27800.67 | 3075.25 |
| ASV_1402 | Papiliotrema | F | 89 | 0.67 | 1.15 |
| ASV_1402 | Papiliotrema | W | 0 | 0 | 0 |
| ASV_1402 | Papiliotrema | W | 10 | 26700 | 9748.4 |
| ASV_1402 | Papiliotrema | W | 18 | 0.67 | 1.15 |
| ASV_1402 | Papiliotrema | W | 49 | 27094.33 | 9334.5 |
| ASV_1402 | Papiliotrema | W | 89 | 1.67 | 2.89 |
| ASV_2859 | Aspergillus | F | 0 | 6751.33 | 6975.58 |
| ASV_2859 | Aspergillus | F | 10 | 23120.33 | 5148.52 |
| ASV_2859 | Aspergillus | F | 18 | 18691.67 | 5562.24 |
| ASV_2859 | Aspergillus | F | 49 | 13048.33 | 3511.84 |
| ASV_2859 | Aspergillus | F | 89 | 6188.67 | 5044.12 |
| ASV_2859 | Aspergillus | W | 0 | 33217 | 3164.19 |
| ASV_2859 | Aspergillus | W | 10 | 12534.67 | 2401.33 |
| ASV_2859 | Aspergillus | W | 18 | 35473 | 2264.36 |
| ASV_2859 | Aspergillus | W | 49 | 12968 | 7452.55 |
| ASV_2859 | Aspergillus | W | 89 | 39216.33 | 4123.6 |
| ASV_3119 | Aspergillus | F | 0 | 34855.67 | 10006.77 |
| ASV_3119 | Aspergillus | F | 10 | 4166.33 | 3230.87 |
| ASV_3119 | Aspergillus | F | 18 | 19527.67 | 11131.19 |
| ASV_3119 | Aspergillus | F | 49 | 5223 | 4878.1 |
| ASV_3119 | Aspergillus | F | 89 | 15868.33 | 8909.13 |
| ASV_3119 | Aspergillus | W | 0 | 13166 | 4288.14 |
| ASV_3119 | Aspergillus | W | 10 | 2908 | 3580.31 |
| ASV_3119 | Aspergillus | W | 18 | 12509.67 | 2453.15 |
| ASV_3119 | Aspergillus | W | 49 | 15104.33 | 13159.17 |
| ASV_3119 | Aspergillus | W | 89 | 12021.67 | 2341.46 |

T = Treatment; F = Filler leaves; W = Wrapper leaves. 0-10-18-49-89 indicates different time point (days).

Table S7: Heatmap correlation analysis between bacterial phyla and environmental factors.

|  | RS | TS | TA | TN | P | Cl | TE | TALC | TAC | TALD | TK | TH |
| --- | --- | --- | --- | --- | --- | --- | --- | --- | --- | --- | --- | --- |
| Firmicutes | -0.37 | -0.41 | -0.21 | -0.63 | 0.45 | 0.05 | -0.18 | -0.35 | 0.02 | -0.30 | -0.65 | 0.75 |
| Proteobacteria | 0.37 | 0.41 | 0.21 | 0.63 | -0.45 | -0.05 | 0.18 | 0.35 | -0.02 | 0.30 | 0.65 | -0.75 |
| Cyanobacteria | 0.63 | 0.49 | 0.32 | 0.01 | 0.24 | -0.40 | -0.18 | 0.38 | -0.15 | 0.01 | -0.26 | -0.15 |
| Bacteroidetes | -0.62 | -0.66 | 0.21 | 0.33 | 0.05 | 0.56 | 0.71 | -0.50 | 0.67 | 0.61 | 0.41 | 0.13 |
| unclassified_Bacteria | 0.04 | -0.08 | -0.04 | -0.29 | 0.44 | -0.12 | -0.03 | -0.26 | -0.42 | -0.38 | -0.04 | 0.35 |
| Tenericutes | -0.23 | -0.33 | 0.44 | 0.16 | -0.07 | 0.58 | 0.23 | -0.17 | 0.07 | 0.01 | 0.11 | 0.46 |
| Verrucomicrobia | 0.14 | 0.30 | -0.12 | 0.36 | -0.50 | -0.03 | 0.05 | 0.21 | 0.02 | 0.24 | 0.30 | -0.54 |
| Fusobacteria | -0.22 | -0.23 | 0.31 | 0.04 | -0.14 | 0.40 | 0.25 | 0.04 | -0.01 | -0.11 | -0.22 | 0.08 |
| Actinobacteria | -0.24 | -0.32 | 0.02 | -0.24 | 0.33 | 0.16 | 0.18 | -0.27 | 0.37 | 0.03 | -0.33 | 0.60 |
| Acidobacteria | 0.06 | 0.16 | 0.10 | -0.11 | -0.37 | 0.22 | -0.12 | 0.22 | 0.03 | -0.30 | -0.54 | 0.32 |

RS = Reducing sugar; TS = Total sugars; TA = Total Acids; TN = Total nitrogen; P = Phosphorous; Cl = Chlorine; TE = Total esters; TALC = Total Alcohols;

TAC = Total Acids; TALD = Total Aldehyde; TK =Total Ketones; TH = Total Heterocycles.

Table S8. Heatmap correlation analysis between fungal genera and environmental factors.

| Fungi | RS | TS | TS | TN | P | Cl | TE | TALC | TAC | ALD | TK | TH |
| --- | --- | --- | --- | --- | --- | --- | --- | --- | --- | --- | --- | --- |
| Aspergillus | 0.33 | 0.23 | -0.14 | -0.60 | 0.31 | -0.34 | -0.57 | 0.36 | 0.19 | -0.19 | -0.85 | 0.44 |
| Sampaiozyma | -0.16 | -0.23 | 0.61 | 0.59 | -0.02 | 0.51 | 0.81 | -0.21 | -0.01 | 0.64 | 0.50 | -0.29 |
| Papiliotrema | -0.10 | 0.02 | -0.20 | 0.31 | -0.34 | -0.03 | 0.10 | -0.22 | -0.17 | -0.10 | 0.60 | -0.42 |
| Exophiala | -0.17 | 0.03 | -0.27 | 0.43 | -0.50 | 0.12 | 0.38 | -0.06 | -0.13 | -0.14 | 0.68 | -0.44 |
| Sagenomella | -0.24 | -0.22 | -0.30 | -0.34 | 0.24 | -0.24 | -0.49 | -0.35 | -0.07 | -0.16 | -0.10 | 0.10 |
| Penicillium | -0.14 | -0.27 | -0.58 | -0.70 | 0.58 | -0.51 | -0.34 | 0.06 | 0.43 | -0.28 | -0.57 | 0.30 |
| Eutypella | -0.42 | -0.26 | -0.05 | -0.16 | 0.02 | 0.29 | 0.03 | -0.08 | -0.21 | -0.37 | -0.11 | 0.42 |
| Didymellaceae | -0.48 | -0.31 | 0.04 | -0.13 | -0.03 | 0.34 | -0.07 | -0.24 | -0.25 | -0.41 | -0.04 | 0.47 |
| Mycosphaerella | -0.21 | -0.24 | 0.23 | 0.15 | 0.14 | 0.19 | 0.52 | 0.01 | -0.05 | 0.27 | 0.20 | -0.37 |
| Neosulcatispora | -0.19 | -0.12 | 0.31 | 0.16 | -0.21 | 0.22 | -0.24 | -0.48 | -0.14 | -0.14 | 0.30 | 0.11 |

RS = Reducing sugar; TS = Total sugars; TA = Total Acids; TN = Total nitrogen; P = Phosphorous; Cl = Chlorine; TE = Total esters; TALC = Total Alcohols;

TAC = Total Acids; TALD = Total Aldehyde; TK =Total Ketones; TH = Total Heterocycles.

Table S9: Spearman’s correlation analysis of environmental factors.

| cor | RS | TS | TA | TN | P | Cl | TE | TALC | TAC | TALD | TK | TH |
| --- | --- | --- | --- | --- | --- | --- | --- | --- | --- | --- | --- | --- |
| RS | 1 | 0.964 | 0.079 | 0.213 | -0.176 | -0.552 | -0.224 | 0.733 | -0.430 | -0.152 | -0.018 | -0.515 |
| TS | 0.964 | 1 | 0.067 | 0.292 | -0.370 | -0.455 | -0.212 | 0.697 | -0.309 | -0.091 | -0.006 | -0.467 |
| TA | 0.079 | 0.067 | 1 | 0.657 | -0.358 | 0.588 | 0.309 | -0.358 | 0.079 | 0.370 | 0.394 | 0.188 |
| TN | 0.213 | 0.292 | 0.657 | 1 | -0.705 | 0.541 | 0.663 | -0.091 | 0.328 | 0.620 | 0.766 | -0.261 |
| P | -0.176 | -0.370 | -0.358 | -0.705 | 1 | -0.552 | -0.224 | -0.127 | -0.297 | -0.224 | -0.382 | -0.006 |
| Cl | -0.552 | -0.455 | 0.588 | 0.541 | -0.552 | 1 | 0.527 | -0.588 | 0.515 | 0.394 | 0.382 | 0.515 |
| TE | -0.224 | -0.212 | 0.309 | 0.663 | -0.224 | 0.527 | 1 | -0.333 | 0.430 | 0.564 | 0.600 | -0.176 |
| TALC | 0.733 | 0.697 | -0.358 | -0.091 | -0.127 | -0.588 | -0.333 | 1 | -0.321 | -0.285 | -0.261 | -0.576 |
| TAC | -0.430 | -0.309 | 0.079 | 0.328 | -0.297 | 0.515 | 0.430 | -0.321 | 1 | 0.770 | 0.042 | 0.321 |
| TALD | -0.152 | -0.091 | 0.370 | 0.620 | -0.224 | 0.394 | 0.564 | -0.285 | 0.770 | 1 | 0.345 | -0.067 |
| TK | -0.018 | -0.006 | 0.394 | 0.766 | -0.382 | 0.382 | 0.600 | -0.261 | 0.042 | 0.345 | 1 | -0.370 |
| TH | -0.515 | -0.467 | 0.188 | -0.261 | -0.006 | 0.515 | -0.176 | -0.576 | 0.321 | -0.067 | -0.370 | 1 |

RS = Reducing sugar; TS = Total sugars; TA = Total Alkaloids; TN = Total nitrogen; P = Phosphorous; Cl = Chlorine; TE = Total esters; TALC = Total Alcohols; TAC = Total Acids; TALD = Total Aldehyde; TK =Total Ketones; TH = Total Heterocycles.

Table S10. List of correlations and P value of filler and wrapper leaves at different time points.

| V1 | V2 | cor | p_value |
| --- | --- | --- | --- |
| F0 | Reducing_sugars | 0.466666667 | 0.205386351 |
| F0 | Total_sugars | 0.333333333 | 0.380713182 |
| F0 | Total_Alkaloids | 0.55 | 0.124976784 |
| F0 | Total_Nitrogen | 0.502096445 | 0.168397435 |
| F0 | Potassium | -0.033333333 | 0.932156736 |
| F0 | Chlorine | -0.05 | 0.898352804 |
| F0 | Total_Esters | 0.25 | 0.516489552 |
| F0 | Total_Alcohols | 0.233333333 | 0.545698778 |
| F0 | Total_Acids | 0 | 1 |
| F0 | Total_Aldehydes | 0.283333333 | 0.460030329 |
| F0 | Total_ketones | 0.483333333 | 0.187469855 |
| F0 | Total_Heterocycles | -0.366666667 | 0.331739801 |
| F10 | Reducing_sugars | 0.733333333 | 0.02455415 |
| F10 | Total_sugars | 0.716666667 | 0.029818036 |
| F10 | Total_Alkaloids | 0.466666667 | 0.205386351 |
| F10 | Total_Nitrogen | 0.418413704 | 0.262381417 |
| F10 | Potassium | -0.3 | 0.432845327 |
| F10 | Chlorine | -0.116666667 | 0.765007943 |
| F10 | Total_Esters | -0.133333333 | 0.732367505 |
| F10 | Total_Alcohols | 0.466666667 | 0.205386351 |
| F10 | Total_Acids | 0 | 1 |
| F10 | Total_Aldehydes | 0.116666667 | 0.765007943 |
| F10 | Total_ketones | 0.033333333 | 0.932156736 |
| F10 | Total_Heterocycles | -0.183333333 | 0.636819812 |
| F18 | Reducing_sugars | 0.6 | 0.087622829 |
| F18 | Total_sugars | 0.55 | 0.124976784 |
| F18 | Total_Alkaloids | 0.6 | 0.087622829 |
| F18 | Total_Nitrogen | 0.543937816 | 0.130054689 |
| F18 | Potassium | -0.283333333 | 0.460030329 |
| F18 | Chlorine | -0.05 | 0.898352804 |
| F18 | Total_Esters | 0.016666667 | 0.966054804 |
| F18 | Total_Alcohols | 0.483333333 | 0.187469855 |
| F18 | Total_Acids | 0.166666667 | 0.66823104 |
| F18 | Total_Aldehydes | 0.333333333 | 0.380713182 |
| F18 | Total_ketones | 0.083333333 | 0.831214098 |
| F18 | Total_Heterocycles | -0.383333333 | 0.308495272 |
| F49 | Reducing_sugars | 0.5 | 0.170470661 |
| F49 | Total_sugars | 0.5 | 0.170470661 |
| F49 | Total_Alkaloids | 0.633333333 | 0.067085787 |
| F49 | Total_Nitrogen | 0.460255075 | 0.212522295 |
| F49 | Potassium | -0.25 | 0.516489552 |
| F49 | Chlorine | 0.033333333 | 0.932156736 |
| F49 | Total_Esters | 0 | 1 |
| F49 | Total_Alcohols | 0.383333333 | 0.308495272 |
| F49 | Total_Acids | 0.016666667 | 0.966054804 |
| F49 | Total_Aldehydes | 0.216666667 | 0.575514896 |
| F49 | Total_ketones | 0.116666667 | 0.765007943 |
| F49 | Total_Heterocycles | -0.2 | 0.605901274 |
| F89 | Reducing_sugars | 0.516666667 | 0.154390121 |
| F89 | Total_sugars | 0.433333333 | 0.243952436 |
| F89 | Total_Alkaloids | 0.55 | 0.124976784 |
| F89 | Total_Nitrogen | 0.316666667 | 0.406397014 |
| F89 | Potassium | -0.3 | 0.432845327 |
| F89 | Chlorine | 0.1 | 0.797971695 |
| F89 | Total_Esters | 0.066666667 | 0.864689785 |
| F89 | Total_Alcohols | 0.416666667 | 0.264586047 |
| F89 | Total_Acids | -0.066666667 | 0.864689785 |
| F89 | Total_Aldehydes | 0.05 | 0.898352804 |
| F89 | Total_ketones | -0.083333333 | 0.831214098 |
| F89 | Total_Heterocycles | -0.2 | 0.605901274 |
| W0 | Reducing_sugars | 0.566666667 | 0.111632988 |
| W0 | Total_sugars | 0.466666667 | 0.205386351 |
| W0 | Total_Alkaloids | 0.55 | 0.124976784 |
| W0 | Total_Nitrogen | 0.493728171 | 0.176759832 |
| W0 | Potassium | -0.383333333 | 0.308495272 |
| W0 | Chlorine | 0.1 | 0.797971695 |
| W0 | Total_Esters | 0.083333333 | 0.831214098 |
| W0 | Total_Alcohols | 0.4 | 0.286105102 |
| W0 | Total_Acids | 0.266666667 | 0.487922275 |
| W0 | Total_Aldehydes | 0.433333333 | 0.243952436 |
| W0 | Total_ketones | 0.083333333 | 0.831214098 |
| W0 | Total_Heterocycles | -0.216666667 | 0.575514896 |
| W10 | Reducing_sugars | 0.533333333 | 0.139226868 |
| W10 | Total_sugars | 0.516666667 | 0.154390121 |
| W10 | Total_Alkaloids | 0.5 | 0.170470661 |
| W10 | Total_Nitrogen | 0.543937816 | 0.130054689 |
| W10 | Potassium | -0.333333333 | 0.380713182 |
| W10 | Chlorine | -0.033333333 | 0.932156736 |
| W10 | Total_Esters | 0.066666667 | 0.864689785 |
| W10 | Total_Alcohols | 0.4 | 0.286105102 |
| W10 | Total_Acids | 0.166666667 | 0.66823104 |
| W10 | Total_Aldehydes | 0.283333333 | 0.460030329 |
| W10 | Total_ketones | 0.116666667 | 0.765007943 |
| W10 | Total_Heterocycles | -0.383333333 | 0.308495272 |
| W18 | Reducing_sugars | 0.533333333 | 0.139226868 |
| W18 | Total_sugars | 0.5 | 0.170470661 |
| W18 | Total_Alkaloids | 0.633333333 | 0.067085787 |
| W18 | Total_Nitrogen | 0.533333333 | 0.139226868 |
| W18 | Potassium | -0.3 | 0.432845327 |
| W18 | Chlorine | 0 | 1 |
| W18 | Total_Esters | 0.266666667 | 0.487922275 |
| W18 | Total_Alcohols | 0.3 | 0.432845327 |
| W18 | Total_Acids | 0.166666667 | 0.66823104 |
| W18 | Total_Aldehydes | 0.433333333 | 0.243952436 |
| W18 | Total_ketones | 0.083333333 | 0.831214098 |
| W18 | Total_Heterocycles | -0.316666667 | 0.406397014 |
| W49 | Reducing_sugars | 0.733333333 | 0.02455415 |
| W49 | Total_sugars | 0.65 | 0.058073058 |
| W49 | Total_Alkaloids | 0.4 | 0.286105102 |
| W49 | Total_Nitrogen | 0.267784771 | 0.486029614 |
| W49 | Potassium | -0.083333333 | 0.831214098 |
| W49 | Chlorine | -0.35 | 0.355819573 |
| W49 | Total_Esters | -0.2 | 0.605901274 |
| W49 | Total_Alcohols | 0.616666667 | 0.07692881 |
| W49 | Total_Acids | -0.166666667 | 0.66823104 |
| W49 | Total_Aldehydes | 0.233333333 | 0.545698778 |
| W49 | Total_ketones | -0.083333333 | 0.831214098 |
| W49 | Total_Heterocycles | -0.483333333 | 0.187469855 |
| W89 | Reducing_sugars | 0.516666667 | 0.154390121 |
| W89 | Total_sugars | 0.433333333 | 0.243952436 |
| W89 | Total_Alkaloids | 0.45 | 0.224216107 |
| W89 | Total_Nitrogen | 0.292889593 | 0.444354696 |
| W89 | Potassium | -0.066666667 | 0.864689785 |
| W89 | Chlorine | -0.166666667 | 0.66823104 |
| W89 | Total_Esters | 0 | 1 |
| W89 | Total_Alcohols | 0.416666667 | 0.264586047 |
| W89 | Total_Acids | 0.25 | 0.516489552 |
| W89 | Total_Aldehydes | 0.316666667 | 0.406397014 |
| W89 | Total_ketones | -0.066666667 | 0.864689785 |
| W89 | Total_Heterocycles | -0.2 | 0.605901274 |

T = Treatment; F = Filler leaves; W = Wrapper leaves. 0-10-18-49-89 indicates different time point (days).

Table S11. List of correlations and P value of filler and wrapper leaves at different time points.

| V1 | V2 | cor | p_value |
| --- | --- | --- | --- |
| F0 | Reducing_sugars | 0.406181197 | 0.244128682 |
| F0 | Total_sugars | 0.522232968 | 0.121502919 |
| F0 | Total_Alkaloids | 0.174077656 | 0.630536076 |
| F0 | Total_Nitrogen | 0.058202525 | 0.873112396 |
| F0 | Potassium | -0.406181197 | 0.244128682 |
| F0 | Chlorine | -0.058025885 | 0.873494892 |
| F0 | Total_Esters | -0.290129427 | 0.416120254 |
| F0 | Total_Alcohols | 0.406181197 | 0.244128682 |
| F0 | Total_Acids | 0.174077656 | 0.630536076 |
| F0 | Total_Aldehydes | 0.058025885 | 0.873494892 |
| F0 | Total_ketones | -0.522232968 | 0.121502919 |
| F0 | Total_Heterocycles | 0.058025885 | 0.873494892 |
| F10 | Reducing_sugars | -0.174077656 | 0.630536076 |
| F10 | Total_sugars | -0.290129427 | 0.416120254 |
| F10 | Total_Alkaloids | 0.406181197 | 0.244128682 |
| F10 | Total_Nitrogen | 0.291012623 | 0.414637469 |
| F10 | Potassium | 0.174077656 | 0.630536076 |
| F10 | Chlorine | 0.174077656 | 0.630536076 |
| F10 | Total_Esters | 0.406181197 | 0.244128682 |
| F10 | Total_Alcohols | -0.058025885 | 0.873494892 |
| F10 | Total_Acids | 0.058025885 | 0.873494892 |
| F10 | Total_Aldehydes | 0.406181197 | 0.244128682 |
| F10 | Total_ketones | 0.290129427 | 0.416120254 |
| F10 | Total_Heterocycles | -0.406181197 | 0.244128682 |
| F18 | Reducing_sugars | -0.058025885 | 0.873494892 |
| F18 | Total_sugars | -0.058025885 | 0.873494892 |
| F18 | Total_Alkaloids | 0.522232968 | 0.121502919 |
| F18 | Total_Nitrogen | 0.407417673 | 0.242556747 |
| F18 | Potassium | -0.174077656 | 0.630536076 |
| F18 | Chlorine | 0.522232968 | 0.121502919 |
| F18 | Total_Esters | 0.290129427 | 0.416120254 |
| F18 | Total_Alcohols | -0.406181197 | 0.244128682 |
| F18 | Total_Acids | 0.406181197 | 0.244128682 |
| F18 | Total_Aldehydes | 0.522232968 | 0.121502919 |
| F18 | Total_ketones | 0.174077656 | 0.630536076 |
| F18 | Total_Heterocycles | 0.522232968 | 0.121502919 |
| F49 | Reducing_sugars | -0.290129427 | 0.416120254 |
| F49 | Total_sugars | -0.174077656 | 0.630536076 |
| F49 | Total_Alkaloids | 0.290129427 | 0.416120254 |
| F49 | Total_Nitrogen | 0.174607574 | 0.629479402 |
| F49 | Potassium | -0.290129427 | 0.416120254 |
| F49 | Chlorine | 0.290129427 | 0.416120254 |
| F49 | Total_Esters | -0.174077656 | 0.630536076 |
| F49 | Total_Alcohols | -0.522232968 | 0.121502919 |
| F49 | Total_Acids | -0.058025885 | 0.873494892 |
| F49 | Total_Aldehydes | -0.058025885 | 0.873494892 |
| F49 | Total_ketones | 0.406181197 | 0.244128682 |
| F49 | Total_Heterocycles | 0.174077656 | 0.630536076 |
| F89 | Reducing_sugars | -0.406181197 | 0.244128682 |
| F89 | Total_sugars | -0.406181197 | 0.244128682 |
| F89 | Total_Alkaloids | -0.058025885 | 0.873494892 |
| F89 | Total_Nitrogen | -0.232810099 | 0.517444664 |
| F89 | Potassium | -0.058025885 | 0.873494892 |
| F89 | Chlorine | 0.406181197 | 0.244128682 |
| F89 | Total_Esters | 0.174077656 | 0.630536076 |
| F89 | Total_Alcohols | -0.174077656 | 0.630536076 |
| F89 | Total_Acids | -0.174077656 | 0.630536076 |
| F89 | Total_Aldehydes | -0.522232968 | 0.121502919 |
| F89 | Total_ketones | -0.174077656 | 0.630536076 |
| F89 | Total_Heterocycles | 0.406181197 | 0.244128682 |
| W0 | Reducing_sugars | 0.522232968 | 0.121502919 |
| W0 | Total_sugars | 0.406181197 | 0.244128682 |
| W0 | Total_Alkaloids | 0.058025885 | 0.873494892 |
| W0 | Total_Nitrogen | -0.058202525 | 0.873112396 |
| W0 | Potassium | 0.406181197 | 0.244128682 |
| W0 | Chlorine | -0.522232968 | 0.121502919 |
| W0 | Total_Esters | 0.058025885 | 0.873494892 |
| W0 | Total_Alcohols | 0.058025885 | 0.873494892 |
| W0 | Total_Acids | -0.522232968 | 0.121502919 |
| W0 | Total_Aldehydes | -0.290129427 | 0.416120254 |
| W0 | Total_ketones | 0.058025885 | 0.873494892 |
| W0 | Total_Heterocycles | -0.290129427 | 0.416120254 |
| W10 | Reducing_sugars | 0.174077656 | 0.630536076 |
| W10 | Total_sugars | 0.290129427 | 0.416120254 |
| W10 | Total_Alkaloids | -0.290129427 | 0.416120254 |
| W10 | Total_Nitrogen | 0.523822722 | 0.120169763 |
| W10 | Potassium | -0.522232968 | 0.121502919 |
| W10 | Chlorine | 0.058025885 | 0.873494892 |
| W10 | Total_Esters | 0.522232968 | 0.121502919 |
| W10 | Total_Alcohols | 0.290129427 | 0.416120254 |
| W10 | Total_Acids | 0.290129427 | 0.416120254 |
| W10 | Total_Aldehydes | 0.290129427 | 0.416120254 |
| W10 | Total_ketones | 0.522232968 | 0.121502919 |
| W10 | Total_Heterocycles | -0.522232968 | 0.121502919 |
| W18 | Reducing_sugars | 0.290129427 | 0.416120254 |
| W18 | Total_sugars | 0.174077656 | 0.630536076 |
| W18 | Total_Alkaloids | -0.174077656 | 0.630536076 |
| W18 | Total_Nitrogen | -0.232810099 | 0.517444664 |
| W18 | Potassium | 0.058025885 | 0.873494892 |
| W18 | Chlorine | -0.290129427 | 0.416120254 |
| W18 | Total_Esters | -0.522232968 | 0.121502919 |
| W18 | Total_Alcohols | 0.522232968 | 0.121502919 |
| W18 | Total_Acids | -0.290129427 | 0.416120254 |
| W18 | Total_Aldehydes | -0.406181197 | 0.244128682 |
| W18 | Total_ketones | -0.058025885 | 0.873494892 |
| W18 | Total_Heterocycles | -0.058025885 | 0.873494892 |
| W49 | Reducing_sugars | 0.058025885 | 0.873494892 |
| W49 | Total_sugars | 0.058025885 | 0.873494892 |
| W49 | Total_Alkaloids | -0.406181197 | 0.244128682 |
| W49 | Total_Nitrogen | -0.407417673 | 0.242556747 |
| W49 | Potassium | 0.290129427 | 0.416120254 |
| W49 | Chlorine | -0.406181197 | 0.244128682 |
| W49 | Total_Esters | -0.406181197 | 0.244128682 |
| W49 | Total_Alcohols | 0.174077656 | 0.630536076 |
| W49 | Total_Acids | -0.406181197 | 0.244128682 |
| W49 | Total_Aldehydes | -0.174077656 | 0.630536076 |
| W49 | Total_ketones | -0.290129427 | 0.416120254 |
| W49 | Total_Heterocycles | -0.174077656 | 0.630536076 |
| W89 | Reducing_sugars | -0.522232968 | 0.121502919 |
| W89 | Total_sugars | -0.522232968 | 0.121502919 |
| W89 | Total_Alkaloids | -0.522232968 | 0.121502919 |
| W89 | Total_Nitrogen | -0.523822722 | 0.120169763 |
| W89 | Potassium | 0.522232968 | 0.121502919 |
| W89 | Chlorine | -0.174077656 | 0.630536076 |
| W89 | Total_Esters | -0.058025885 | 0.873494892 |
| W89 | Total_Alcohols | -0.290129427 | 0.416120254 |
| W89 | Total_Acids | 0.522232968 | 0.121502919 |
| W89 | Total_Aldehydes | 0.174077656 | 0.630536076 |
| W89 | Total_ketones | -0.406181197 | 0.244128682 |
| W89 | Total_Heterocycles | 0.290129427 | 0.416120254 |

T = Treatment; F = Filler leaves; W = Wrapper leaves. 0-10-18-49-89 indicates different time point (days).
